# Congenital Syphilis in Brazil: Municipal Antenatal Care, Primary Health Care Capacity, and Territorial Inequalities, 2015–2024

**DOI:** 10.1007/s10995-026-04329-1

**Published:** 2026-09-19

**Authors:** Alice Ramos-Silva, Camila de Melo Carvalho Nascimento, Luis Phillipe Nagem Lopes, Fabiana Rabe Carvalho

**Affiliations:** 1 Faculty of Pharmacy, Universidade Federal do Rio de Janeiro, Rio de Janeiro, RJ Brazil; 2 Postgraduate Program in Medical Sciences, Faculty of Medicine, Universidade Federal Fluminense, Niterói, RJ Brazil; 3 Instituto de Medicina Social, Universidade do Estado do Rio de Janeiro, Rio de Janeiro, RJ Brazil; 4 Postgraduate Program in Pathology, Faculty of Medicine, Universidade Federal Fluminense, Niterói, RJ Brazil

**Keywords:** Congenital syphilis, Antenatal care, Primary health care, Spatial epidemiology, Health equity

## Abstract

**Objectives:**

To describe spatial patterns of gestational (GS) and congenital syphilis (CS) in Brazil and assess municipal antenatal care, sociodemographic, and primary-care correlates of CS notifications after accounting for GS burden.

**Methods:**

We conducted a nationwide ecological study using routinely collected SINAN, SINASC, and CNES data (2015–2024). Spatial analyses used empirical Bayes smoothing, Global Moran’s I, and LISA. Associations were estimated with a Bayesian negative-binomial spatiotemporal model including municipal random effects, annually replicated Besag fields, a first-order random walk, and an estimated coefficient for GS burden. Sensitivity analyses evaluated formulations, adjacency, priors, and age composition.

**Results:**

Spatial analyses included 55,699 municipality-years from 5,570 municipalities; the primary model included 28,436 observations from 3,625 municipalities. CS showed recurrent high–high clusters in the North and Northeast. Per 10-percentage-point increase, fewer than seven antenatal visits (adjusted ratio 1.10, 95% CrI 1.08–1.11) and births to women recorded as Black or Brown (1.07, 1.06–1.08) were positively associated with expected CS notifications, whereas low maternal education was inversely associated (0.93, 0.92–0.95). Each additional registered Family Health Strategy team per 10,000 population was inversely associated (0.89, 0.87–0.91), although this estimate was model-sensitive. GS elasticity was 0.72 (0.71–0.74); estimates were stable under KNN-5.

**Conclusions for Practice:**

CS remained spatially concentrated, and municipal antenatal-care utilization, sociodemographic composition, and primary-care capacity were associated with expected CS notifications after accounting for GS burden. These ecological findings may help identify territories where care delivery, primary-care capacity, and surveillance warrant closer assessment. They do not estimate individual transmission risk or establish causal effects.

**Supplementary Information:**

The online version contains supplementary material available at https://doi.org/10.1007/s10995-026-04329-1.

## Significance

What Is Already Known?

What Does This Study Add?

Congenital syphilis is largely preventable through timely diagnosis and treatment during pregnancy but remains a persistent public health problem in Brazil, with marked geographic inequalities.

This municipal study identified recurrent congenital syphilis clusters in the North and Northeast. Associations with antenatal-care utilization, social composition, and registered primary-care capacity persisted after additional age adjustment, although the capacity association was sensitive to model specification. These findings support territorial assessment of care and surveillance, not individual transmission-risk estimation or causal inference.

**Supplementary Information:**

The online version contains supplementary material available at https://doi.org/10.1007/s10995-026-04329-1.

## Introduction

Syphilis during pregnancy remains a major public health concern because untreated or inadequately treated infection can cause fetal infection, miscarriage, stillbirth, preterm birth, neonatal death, and congenital syphilis (CS) (Gilmour & Walls, 2023; Korenromp et al., 2019). CS is largely preventable through timely diagnosis, repeat antenatal testing, adequate penicillin treatment, partner management, and continuity of follow-up (Carvalho et al., 2023; Gilmour & Walls, 2023). At the population level, persistent CS can therefore signal gaps in the timely delivery, continuity, and equity of maternal health services (Costa et al., 2024).

Brazil has maintained a high CS burden despite broad antenatal-care coverage and free diagnosis and treatment through the Unified Health System. National surveillance indicates that CS incidence increased from 6.6 per 1,000 live births in 2015 to 9.6 per 1,000 in 2024 (Brazilian Ministry of Health, 2025). Interpretation of this period must also consider changes in prevention and surveillance: benzathine penicillin was maintained in the SUS for prevention of CS, rapid testing and repeated antenatal screening were expanded, surveillance definitions were revised, and primary-care performance monitoring increasingly incorporated antenatal and syphilis-testing indicators within Brazil’s Family Health Strategy-centered system. The COVID-19 period further affected health-service use and case ascertainment, so notification trends cannot be interpreted as changes in prevention performance alone (Sentís et al., 2021).

Conceptually, municipal social conditions may shape primary-care capacity and antenatal-care utilization, which influence opportunities for testing, diagnosis, treatment, partner management, and follow-up; observed CS notifications are also shaped by surveillance completeness and ascertainment. Our indicators capture selected components of this pathway: maternal education and race/skin color describe municipal social composition, fewer than seven antenatal visits measure recorded utilization, and FHS team density represents registered primary-care capacity rather than realized access or quality. Brazilian studies have documented geographic and ethnoracial inequalities in GS and CS, but evidence on these municipal contextual correlates remains limited (Lopes de Oliveira et al., 2023; Paixão et al., 2023; Pinheiro et al., 2026). We therefore described the spatial distribution of GS and CS across Brazilian municipalities from 2015 to 2024 and assessed municipal antenatal-care, sociodemographic, and primary-care correlates of CS notifications after accounting for GS notification burden.

## Methods

### Study Design and Data Sources

We conducted a nationwide ecological study using municipality-year as the analytical unit and a fixed panel of all 5,570 Brazilian municipalities from 2015 to 2024, with reporting guided by the RECORD statement (Benchimol et al., 2015) and SAMPL recommendations (Lang & Altman, 2015) (Online Resource 1). GS and CS notifications were obtained from the Notifiable Diseases Information System (SINAN); live births and characteristics of women giving birth from the Live Birth Information System (SINASC); and Family Health Strategy (FHS) team records from monthly National Registry of Health Establishments (CNES-EP) files. FHS is Brazil’s community-based primary health care model, delivered by multidisciplinary teams responsible for defined territories and populations.

Each source was processed separately, municipal identifiers were harmonized, and records were aggregated by municipality of residence and calendar year before merging. Notification counts were coded as zero only when the corresponding annual source file was available; thus, zero indicated no recorded notification rather than absence of infection. FHS records were deduplicated by team, municipality, and month. Population denominators came from IBGE annual estimates, the 2022 Census, and interpolation for 2023. The complete panel supported descriptive temporal and spatial analyses, whereas the prespecified eligible subset was used to assess municipal correlates of CS notifications after accounting for GS notification burden.

### Outcomes and Contextual Covariates

For descriptive spatial analyses, annual GS detection and CS incidence rates were calculated per 1,000 live births; municipality-years without a positive live-birth denominator were excluded. Regional rates were based on summed notifications and live births.

For inferential analyses, the outcome was the annual number of CS notifications per municipality-year, with GS notification burden entered on the log scale using an estimated coefficient. Because GS and CS records were not linked at the pregnancy–infant level, estimates represented contextual associations between notification processes, not individual transmission probabilities or direct measures of prevention failure.

The four prespecified municipal covariates were the proportions of live births to women with fewer than seven antenatal visits, ≤ 7 completed years of schooling, and Black or Brown race/skin color (*preta* or *parda*), and FHS team density. Proportions used variable-specific valid-record denominators. The antenatal-care threshold reflected the stable SINASC categories of none, 1–3, 4–6, and ≥ 7 visits and was interpreted as recorded utilization, not access or quality; it did not capture care initiation, gestational age at delivery, or clinical content. Maternal and partner treatment variables were summarized descriptively but were not included as primary covariates because they were recorded only among notified GS cases, could not be linked to subsequent CS records, and represent downstream care-process measures conditional on case detection.

FHS capacity was measured as the annual mean number of registered teams per 10,000 inhabitants and did not represent effective coverage, realized access, or care quality. Proportions were scaled per 10-percentage-point increase and FHS density per additional team per 10,000 inhabitants, using the same centering constants across models.

### Spatial Analysis

Annual GS and CS rates were smoothed using global empirical Bayes estimation. First-order queen contiguity was used as the primary spatial specification because it defines municipal neighbors through shared boundaries or vertices while preserving the geographic adjacency structure of the areal data. Global Moran’s I and Local Indicators of Spatial Association (LISA) were calculated from the smoothed rates using row-standardized weights. Annual Global Moran’s I was evaluated with 999 permutations. LISA classified municipalities as high-high, low-low, high-low, low-high, or nonsignificant at an unadjusted two-sided *p* < 0.05 and was interpreted descriptively because of multiple local testing.

### Primary Bayesian Negative-Binomial Spatiotemporal Model

The primary inferential analysis included municipality-years with ≥ 100 live births, ≥ 1 GS notification, and complete data for the four prespecified covariates, yielding 28,436 municipality-years from 3,625 municipalities. Municipality-years with CS notifications exceeding GS notifications were retained.

Let C__it_ and G__it_ denote annual CS and GS notifications in municipality i and year t. We fitted a Bayesian negative-binomial spatiotemporal model:

$$ C\_it\sim NegBin\left( {\mu \_it,{\text{ }}k} \right) $$

$$ log\left( {\mu \_it} \right) = \alpha + \delta {\text{ }}log\left( {G\_it} \right) + x\_it^{T} \beta + u\_i + s\_it + \gamma \_t $$

$$ Var\left( {C\_it{\text{ }}|{\text{ }}\mu \_it,{\text{ }}k} \right) = \mu \_it + \mu \_it^{2} /k $$

The coefficient δ was estimated rather than fixed at 1. Municipality-years with zero GS notifications were excluded because log(G__it_) was undefined. These observations were not assigned an artificial GS value because zero notifications may reflect either true absence of reported disease or incomplete detection and reporting. The vector x__it_ contained the four prespecified covariates; *u*__i_ was a municipality-specific random intercept, *s*__it_ an annually replicated Besag spatial effect based on queen contiguity with shared precision, and γ__t_ a first-order random walk (RW1). The municipality-specific random intercept was shared across annual observations from the same municipality, thereby accounting for within-municipality dependence over time. Besag effects used scaling, sum-to-zero constraints, and adjustment for disconnected graph components. Extra-Poisson variation was modeled through the negative-binomial dispersion parameter.

Models were fitted with INLA. Regression coefficients had N(0,2.5²) priors and the intercept N(0,5²); random-effect standard deviations used penalized-complexity priors with *P*(*SD* > 1) = 0.01. In implementation, GS was supplied as an exposure and a centered log(GS) term with coefficient λ ~ N(0,1) was added, giving total elasticity δ = 1 + λ. The negative-binomial size parameter used INLA variant 0 with a pc.mgamma prior parameter of 7. Exponentiated covariate coefficients were reported as adjusted ratios of expected CS notifications with 95% credible intervals (CrIs). RW1 contrasts were reported relative to 2015, and δ was reported directly with its 95% CrI.

### Model-Development and Sensitivity Analyses

Sensitivity analyses separated outcome formulation, sample restriction, and GS scaling (Online Resource 2, Table S1). L1H, a grouped-binomial logit-normal model, included 26,513 municipality-years from 3,622 municipalities meeting the primary eligibility criteria and CS ≤ GS, with municipal, annually replicated Besag, RW1, and municipality-year random effects. NB1 used a negative-binomial likelihood and a unit log(GS) offset on this restricted sample. NB2 retained that specification but included 1,923 additional CS > GS observations; NB3 used the same expanded sample and estimated the log(GS) coefficient.

A common queen graph of 3,625 municipalities was used across models. CPO comparisons were limited to identical samples (L1H versus NB1; NB2 versus NB3); DIC and WAIC were not compared across different samples. NB3 was selected because it retained CS > GS, allowed nonunit GS scaling, and improved prediction and aggregate calibration versus NB2. Temporal contrasts are reported in Table S2.

Spatial sensitivity replaced queen contiguity with a symmetrized KNN-5 graph using projected municipality point-on-surface coordinates (Table S3). Prior sensitivity varied regression, GS-elasticity, and latent-effect priors while retaining the negative-binomial dispersion prior (Table S4). An additional model included SINASC proportions of births to women aged < 20 and ≥ 35 years, per 10-percentage-point increase among records with valid age, retaining education, and all other model components (Table S5). Collinearity was assessed using Pearson/Spearman correlations, variance inflation factors (VIFs), and fixed-design rank, including log(GS) (Table S6).

### Model Assessment, Missing Data, Software, and Ethics

Model assessment examined estimation warnings, CPOs, observed-versus-fitted calibration, Pearson-type residuals, and posterior predictive checks of total notifications, zeros, dispersion, and extremes (Table S7). Annual residual Moran’s I used 999 permutations and Holm correction; pooled lag-1 correlation summarized residual temporal dependence.

Quality checks assessed source coverage, municipal identifiers, denominators, missing fields, and discordant CS/GS counts. Sample selection, aggregate availability, and SINASC source-record completeness by year and region are reported in Table S8. Missing, unknown, or invalid field values were excluded from variable-specific denominators; no imputation was performed. Analyses used R 4.6.1; scripts are provided in Online Resource 3. The study used publicly available secondary data without direct personal identifiers. Under Brazilian National Health Council Resolution No. 466/2012, ethics committee review and individual informed consent were not required.

## Results

### Municipality-Year Panel

Of 55,700 municipality-years, 55,699 had positive live-birth denominators; 22,111 (39.7%) had fewer than 100 live births. The primary NB3 sample comprised 28,436 observations from 3,625 municipalities, including 1,923 (6.8%) with CS > GS. All primary covariates and age indicators were available; no additional observations were excluded for aggregate missingness. Among 25,305,992 SINASC birth records within this sample, nonvalid information comprised 0.003% for age, 1.11% for education, 3.42% for race/skin color, and 0.52% for antenatal visits (Table S8).

Table S8 presents national and regional counts, rates, CS/GS notification ratios, and covariate distributions by year. In 2024, GS detection ranged from 27.35 per 1,000 live births in the Northeast to 46.74 in the Southeast; CS incidence ranged from 8.30 in the Midwest to 10.75 in the Southeast.

Absolute national and region-year GS and CS notification counts, GS detection rates, CS incidence rates, and CS notifications per 100 reported GS notifications are presented in Table S8, Panel A, allowing assessment of regional temporal trends.

### Reported Gestational Syphilis Notifications

Between 2015 and 2024, 651,140 GS notifications were recorded nationally. The largest proportions occurred among women aged 20–24 years (34.8%). Third-trimester diagnosis occurred in 28.9% of GS notifications, and adequate maternal treatment with documented partner treatment was recorded in 30.6% (Table 1).

**Table 1:** Sociodemographic and clinical characteristics of gestational syphilis cases in Brazil, 2015–2024 (*N* = 651,140)

Characteristic	*n* (%)
Age range (years)	
< 15	6,405 (1.0)
15–19	144,586 (22.2)
20–24	226,364 (34.8)
25–29	144,394 (22.2)
30–34	77,205 (11.9)
35–39	39,217 (6.0)
40–44	11,810 (1.8)
45–49	966 (0.1)
Unknown	193 (0.0)
Skin color	
White	187,509 (28.8)
Black	79,741 (12.2)
Brown	335,790 (51.6)
Asian	6,553 (1.0)
Indigenous	3,015 (0.5)
Unknown	38,532 (5.9)
Education (years)	
≤ 9 years	203,384 (31.2)
> 9 years	277,077 (42.6)
Unknown	170,679 (26.2)
Diagnosis by trimester of pregnancy	
1 st trimester	278,551 (42.8)
2nd trimester	148,875 (22.9)
3rd trimester	188,106 (28.9)
Unknown	35,608 (5.5)
Region of residence	
Southeast	300,927 (46.2)
Northeast	135,106 (20.7)
South	97,144 (14.9)
North	64,636 (9.9)
Central-West	53,327 (8.2)
Treatment	
Adequate maternal treatment without documented partner treatment	385,831 (59.3)
Adequate maternal treatment with documented partner treatment	199,319 (30.6)
Inadequate	44,226 (6.8)
Unknown	21,764 (3.3)

### Spatial Distribution

**Fig. 1:**
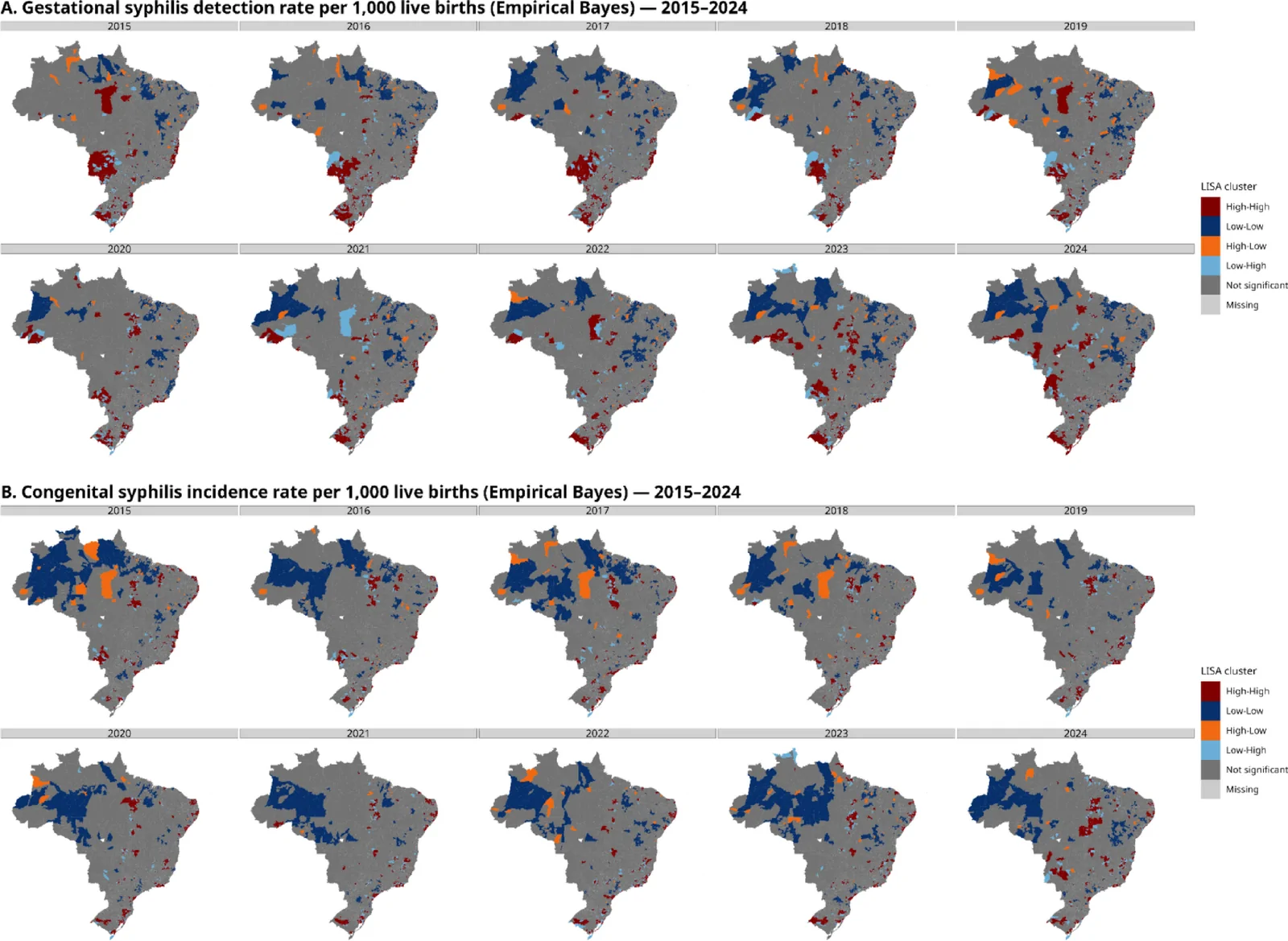
Local spatial clusters of gestational and congenital syphilis in Brazil, 2015–2024. **A** shows LISA clusters based on empirical-Bayes-smoothed gestational syphilis detection rates per 1,000 live births, and **B** shows LISA clusters based on empirical-Bayes-smoothed congenital syphilis incidence rates per 1000 live births. First-order queen contiguity and row-standardized spatial weights were used. Municipalities were classified as high-high, low-low, high-low, low-high, not significant, or missing

**Fig. 2:**
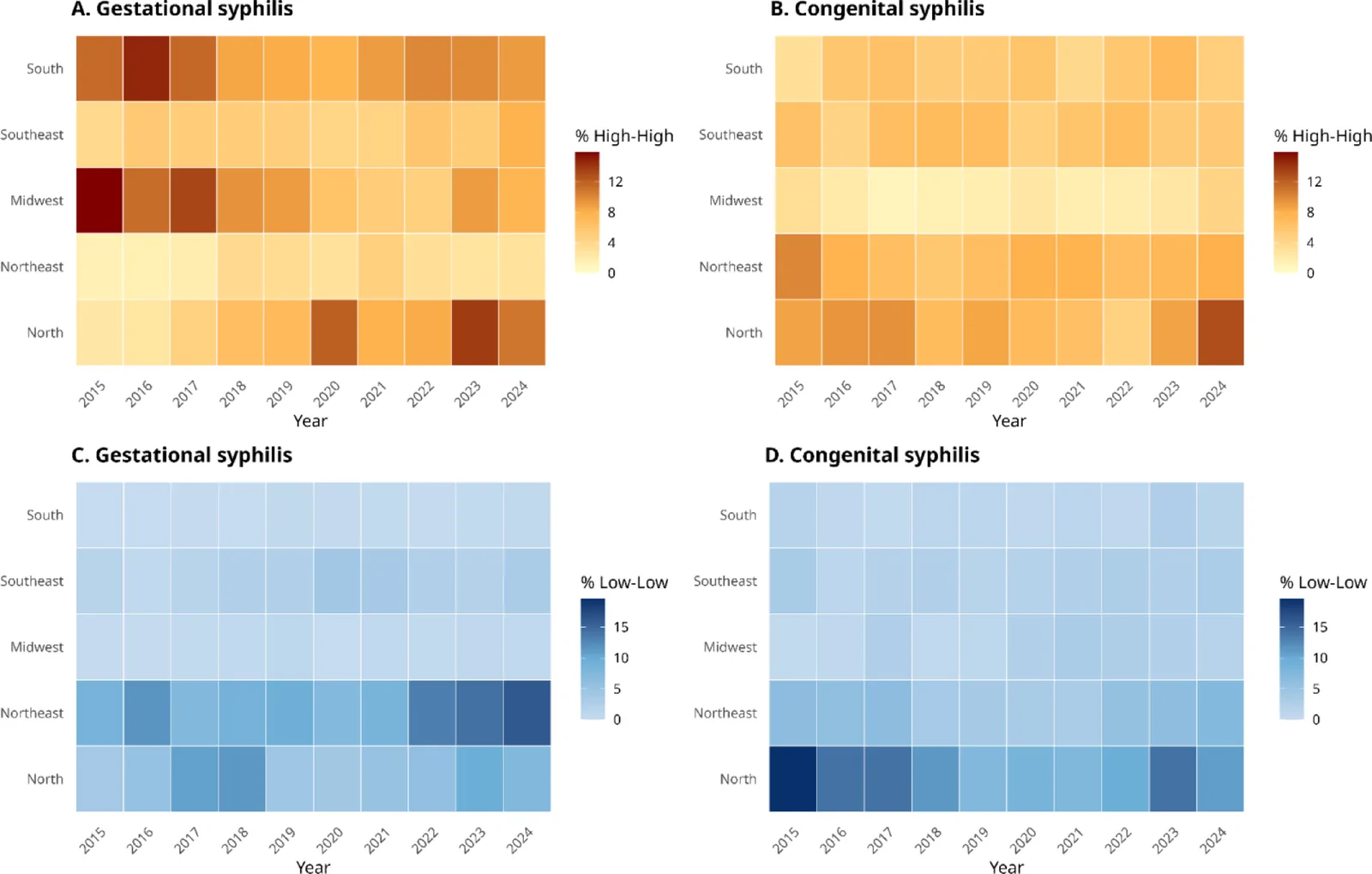
Percentage of municipality-year observations classified as high-high or low-low LISA clusters by Brazilian region and year, 2015–2024. **A** and **C** show gestational syphilis high-high and low-low clusters, respectively; **B** and **D** show congenital syphilis high-high and low-low clusters. Values are the percentage of municipalities within each macro-region classified in the corresponding LISA category in each year

Both GS and CS exhibited spatial clustering throughout the study period. Using global empirical Bayes-smoothed rates and queen-contiguity weights, annual Global Moran’s I ranged from 0.223 to 0.308 for GS and from 0.291 to 0.357 for CS; all annual permutation tests yielded *p* = 0.001 (Table S9). LISA analyses identified recurrent high-high CS clusters, particularly in the North and Northeast, although their geographic extent varied over time (Fig. 1; Tables S10 and S11). Low-low CS clustering was most prominent in the North and also occurred in the Northeast. For GS, high-high clusters were proportionally most frequent in the South and Midwest over the aggregated study period, whereas low-low clusters were concentrated mainly in the Northeast and North (Fig. 1; Tables S12 and S13). Regional heatmaps showed temporal variation in high-high and low-low classifications for both outcomes (Fig. 2).

### Municipal Correlates of Congenital Syphilis Notifications

In the primary NB3 model, a 10-percentage-point higher proportion of live births to women with fewer than seven antenatal visits was associated with higher expected CS notifications (adjusted ratio 1.10, 95% CrI 1.08–1.11), as was a higher proportion to women recorded as Black or Brown (1.07, 1.06–1.08). A higher proportion to women with ≤ 7 completed years of schooling was inversely associated (0.93, 0.92–0.95), and each additional registered FHS team per 10,000 inhabitants was also inversely associated (0.89, 0.87–0.91) (Table 2).

**Table 2:** Municipal correlates of congenital syphilis notifications after accounting for gestational syphilis notification burden, Brazil, 2015–2024

Variable	Adjusted ratio of expected CS notifications (95% CrI)
Municipal characteristics	
Live births to women with fewer than seven antenatal visits (+ 10% points)	1.097 (1.080–1.115)
Live births to women with ≤ 7 years of schooling (+ 10% points)	0.934 (0.915–0.952)
Live births to women recorded as Black or Brown (+ 10% points)	1.070 (1.056–1.085)
Registered Family Health Strategy team density (+ 1 team per 10,000 population)	0.889 (0.872–0.906)
Calendar year (RW1 temporal contrast; 2015 reference)	
2015	Reference
2016	0.981 (0.943–1.022)
2017	0.943 (0.909–0.985)
2018	0.905 (0.865–0.945)
2019	0.864 (0.828–0.906)
2020	0.792 (0.755–0.830)
2021	0.828 (0.791–0.867)
2022	0.809 (0.767–0.852)
2023	0.799 (0.754–0.848)
2024	0.816 (0.771–0.864)

Estimates are posterior medians and 95% credible intervals (CrIs) from NB3, a Bayesian negative-binomial spatiotemporal model fitted to 28,436 municipality-year observations from 3,625 municipalities with ≥ 100 live births and ≥ 1 gestational syphilis (GS) notification. Municipality-years with congenital syphilis (CS) notifications exceeding GS notifications were retained. The model included municipality-level unstructured effects, annual Besag spatial fields with shared precision, and a first-order random-walk temporal effect. GS notification burden was modeled with an estimated total log-log elasticity rather than a fixed unit-offset assumption (δ = 0.722, 95% CrI 0.706–0.738). Ratios describe relative expected CS notification frequency for the specified covariate increment; they are not individual transmission risks or causal effects. Calendar-year estimates are posterior contrasts of the national RW1 temporal component relative to 2015 and do not represent crude annual notification ratios. FHS, Family Health Strategy

Posterior contrasts of the national temporal component were compatible with the 2015 reference in 2016 (0.98, 95% CrI 0.94–1.02) and lower than 2015 from 2017 onward. In the primary model, the lowest posterior median occurred in 2020 (0.79, 0.76–0.83); the 2024 estimate remained below 2015 (0.82, 0.77–0.86) (Table 2).

### Sensitivity Analyses and Model Diagnostics

Across model formulations, antenatal-care, education, and race/skin-color associations retained their directions, whereas FHS was sensitive to GS scaling (Table S1). Estimated GS elasticity was 0.722 (95% CrI 0.706–0.738), with improved prediction and calibration versus NB2 and no Holm-significant residual Moran’s I under queen contiguity. Estimates were similar under KNN-5, despite modest negative residual autocorrelation in 2019, 2022, and 2023 (Table S3), and under alternative priors (Table S4).

Age adjustment preserved the four association directions and 95% credible-interval interpretations; education attenuated from 0.934 to 0.945 (95% CrI 0.925–0.964). The lowest temporal median differed by specification (Table S5). The age-augmented municipality-year fixed design had full rank and VIFs of 1.38–3.95 (Table S6). Both reference and age-adjusted models underpredicted zeros and overpredicted maximum counts in posterior predictive checks (Table S7).

## Discussion

In this nationwide municipality-year study, CS showed stronger and more persistent spatial clustering than GS, with recurrent high-high clusters in the North and Northeast. After accounting for GS notification burden, higher municipal proportions of live births to women with fewer than seven antenatal visits and to women recorded as Black or Brown were associated with higher expected CS notifications. Low maternal education was inversely associated, whereas greater registered FHS density showed an inverse but model-sensitive association. These findings indicate that CS notifications are patterned by antenatal-care utilization, social composition, and primary-care capacity beyond maternal syphilis notification burden alone.

The association with fewer than seven antenatal visits reinforces the importance of antenatal-care engagement in CS prevention. This indicator measured recorded utilization rather than quality of care, but its positive association is consistent with prevention requiring timely diagnosis, repeat testing when indicated, adequate penicillin treatment, and continuity of follow-up during pregnancy (Carvalho et al., 2023; Fanella et al., 2024; Gilmour & Walls, 2023; Salomè et al., 2024). Brazilian ecological evidence has likewise linked CS burden to prenatal-care indicators and access to testing (Costa et al., 2024; Eller et al., 2025). Only 30.6% of GS notifications were classified as having adequate maternal treatment with documented partner treatment, highlighting weaknesses beyond service contact. Untreated partners can permit reinfection, and Brazilian evidence links ineffective maternal treatment and absent partner treatment to CS (Swayze et al., 2022; Fernandes et al., 2026). Thus, visit counts should not be treated as an isolated intervention target; timing, diagnostic content, treatment, partner management, and follow-up remain central.

The positive association with the municipal proportion of live births to women recorded as Black or Brown should not be interpreted as biological susceptibility. Race/skin color in Brazil is a social marker shaped by structural racism, socioeconomic inequality, and unequal access to timely and continuous care. National record-linkage evidence has documented substantial ethnoracial inequalities in maternal and congenital syphilis, including intersections with maternal education (Paixão et al., 2023). Our findings add a territorial dimension by showing that this association persisted after adjustment for antenatal-care utilization, FHS density, temporal variation, spatial structure, and GS notification burden.

The inverse association with low maternal education requires particular caution. Its direction was consistent across revised model specifications but contrasts with individual-level evidence showing greater syphilis burden among socioeconomically disadvantaged and less-educated women (Paixão et al., 2023). Education was a municipality-level compositional measure entered simultaneously with antenatal utilization, race/skin color, FHS capacity, spatial structure, and GS burden. The adjusted ecological coefficient may therefore reflect the joint distribution of these characteristics, differential ascertainment, or unmeasured municipal processes rather than a protective effect of lower schooling. This illustrates why aggregate coefficients should not be translated into individual-level effects.

Registered FHS team density was inversely associated with expected CS notifications in NB3 and changed little under KNN-5 adjacency. However, this association was more sensitive to GS scaling: under NB2, in which GS was constrained as a unit offset, its credible interval included the null. The FHS result should therefore be interpreted as a model-sensitive contextual association rather than evidence that increasing team density would itself reduce CS. Brazilian studies support the relevance of primary care for syphilis detection and antenatal management (Saraceni & Miranda, 2012; Eller et al., 2025), but our measure represents registered capacity rather than effective coverage, timely access, staffing continuity, or quality.

The relationship between GS and CS surveillance is also informative. Some municipality-years recorded CS without GS, and others recorded more CS than GS. The estimated GS elasticity of 0.722 (95% CrI 0.706–0.738) indicated a subproportional association between the notification series, supporting treatment of GS as correlated municipal notification burden rather than assuming a fixed CS: GS proportion. Differences in ascertainment, notification timing, completeness, and local surveillance practice may contribute to this discordance. Brazilian SINAN evaluations have documented improving completeness but persistent heterogeneity across variables, regions, and population groups (Oliveira et al., 2023). The estimand is therefore a contextual notification-based measure shaped by care processes, ascertainment, completeness, and reporting rather than an individual transmission probability or a direct measure of prevention failure. Temporal contrasts likewise require caution because the COVID-19 pandemic may have affected antenatal-care attendance, syphilis testing and treatment, service utilization, case ascertainment, and reporting; therefore, the 2020–2021 notification gaps and lower temporal estimates should not be interpreted as evidence of improved prevention.

Several limitations should be considered. The ecological design precludes individual-level inference, and GS and CS notifications were aggregated independently without pregnancy–infant linkage. Although aggregate covariates were complete, proportions used valid field values; completeness varied by year and region, particularly for race/skin color, and differential recording could bias associations. Posterior predictive discrepancies in zeros and maxima indicated remaining distributional limitations despite aggregate calibration. Municipality-years with no reported GS could not enter models containing log(GS), including some with reported CS. This restriction may introduce selection bias if municipalities with reported GS differ systematically in surveillance, diagnostic capacity, or underlying disease burden; inferential estimates therefore apply to the eligible notification-based panel. The antenatal-visit indicator did not account for gestational age at delivery or measure care initiation, testing frequency, treatment timeliness, or clinical content. FHS density measured registered teams rather than effective access or quality. The analysis could not account for previous syphilis infection or reinfection, gravidity or parity, HIV status, antenatal testing patterns, or partner treatment at the individual level; residual confounding by unmeasured municipal characteristics remains possible. Future studies using linked individual-level maternal and infant records could examine the care pathways underlying these territorial patterns, including timing of diagnosis and treatment, reinfection, partner management, and continuity of antenatal follow-up.

Strengths include nationwide 10-year coverage, explicit spatiotemporal modeling of repeated municipality-year data, and sensitivity analyses of outcome formulation, GS scaling, sample restriction, and neighborhood definition.

For practice, these findings support territorial prioritization rather than causal ranking of interventions. Persistent clusters may identify settings where antenatal-care pathways and surveillance warrant closer assessment, including timely diagnosis and treatment, repeat testing, partner management, follow-up, and reporting completeness. Model-sensitive FHS and education estimates caution against interpreting individual covariates as isolated intervention effects.

## Conclusions for Practice

CS remained spatially concentrated and was associated with municipal antenatal-care utilization, sociodemographic composition, and registered primary-care capacity after accounting for GS notification burden. These ecological notification-based findings may help identify territories where antenatal-care delivery, primary-care capacity, and surveillance performance warrant closer assessment and prioritization. They do not estimate individual transmission risk or establish causal effects.

## Supplementary Information

Below is the link to the electronic supplementary material.

Supplementary Material 1

Supplementary Material 2

Supplementary Material 3

Supplementary Material 4

Supplementary Material 5

Supplementary Material 6

Supplementary Material 7

Supplementary Material 8

Supplementary Material 9

Supplementary Material 10

Supplementary Material 11

Supplementary Material 12

Supplementary Material 13

Supplementary Material 14

Supplementary Material 15

Supplementary Material 16

Supplementary Material 17

Supplementary Material 18

## Data Availability

The data used in this study are publicly available from Brazilian Ministry of Health information systems and national administrative databases.

## Code Availability

The analytic code is available from the corresponding author upon reasonable request.
