# Supplementary material for: Congenital Syphilis in Brazil: Municipal Antenatal Care, Primary Health Care Capacity, and Territorial Inequalities, 2015–2024
Source: Matern Child Health J. 2026 Sep 19;30(10):1304–13. doi: 10.1007/s10995-026-04329-1 (PMC13672521; doi:10.1007/s10995-026-04329-1)
Supplement: Supplementary file 1 [file 10995_2026_4329_MOESM11_ESM.docx]

**Online Resource 2. Supplementary Tables**

Congenital syphilis in Brazil: municipal antenatal care, primary health care capacity, and territorial inequalities, 2015–2024.

**Table S1. Sensitivity of municipal associations to outcome formulation, exclusion of municipality-years with CS>GS, and treatment of gestational syphilis notification burden.**

**Panel A. Model specification and municipal associations.**

| **Model** | **Outcome / GS specification** | **Municipality-years** | **Municipalities** | **Antenatal visits <7, +10 pp** | **Maternal education ≤7 years, +10 pp** | **Black or Brown women, +10 pp** | **FHS teams, +1 per 10,000** |
| --- | --- | --- | --- | --- | --- | --- | --- |
| L1H | Grouped binomial logit-normal; CS events / GS trials; municipality-years with CS≤GS | 26513 | 3622 | 1.143 (1.116–1.171) | 0.938 (0.911–0.967) | 1.077 (1.057–1.098) | 0.958 (0.933–0.983) |
| NB1 | Negative binomial; GS fixed as exposure/offset; same CS≤GS sample as L1H | 26513 | 3622 | 1.082 (1.066–1.098) | 0.961 (0.943–0.979) | 1.043 (1.031–1.056) | 0.981 (0.965–0.997) |
| NB2 | Negative binomial; GS fixed as exposure/offset; includes municipality-years with CS>GS | 28436 | 3625 | 1.098 (1.081–1.116) | 0.965 (0.946–0.984) | 1.051 (1.037–1.065) | 0.996 (0.979–1.014) |
| NB3 | Negative binomial; flexible log(GS) scaling (estimated total elasticity); includes municipality-years with CS>GS | 28436 | 3625 | 1.097 (1.080–1.115) | 0.934 (0.915–0.952) | 1.070 (1.056–1.085) | 0.889 (0.872–0.906) |

**Panel B. Model scaling and diagnostics.**

| **Model** | **Estimate scale** | **GS scaling** | **Mean -log(CPO)** | **Total calibration error** | **Maximum absolute annual residual Moran's I** | **Holm-significant residual Moran years** | **Residual lag-1 correlation** |
| --- | --- | --- | --- | --- | --- | --- | --- |
| L1H | Conditional OR | GS used as binomial trials | 2.042 | +0.80% | 0.019 | 0 | 0.013 |
| NB1 | Adjusted expected-notification ratio | Elasticity fixed at 1 | 1.738 | +0.97% | 0.029 | 0 | 0.045 |
| NB2 | Adjusted expected-notification ratio | Elasticity fixed at 1 | 1.898 | +2.66% | 0.03 | 0 | 0.026 |
| NB3 | Adjusted expected-notification ratio | Elasticity δ = 0.722 (0.706–0.738) | 1.864 | −0.17% | 0.026 | 0 | 0.03 |

Note. All models use municipality-year aggregate notifications; GS and CS records were not individually linked. L1H is a harmonized re-estimation of the binomial logit-normal model on the CS≤GS sample and reports conditional odds ratios. NB1, NB2, and NB3 are negative-binomial spatiotemporal models and report adjusted ratios of expected CS notifications. NB1 and NB2 differ by inclusion of municipality-years with CS>GS; NB2 and NB3 use the same expanded sample and differ in whether the total log-log elasticity of GS is fixed at 1 or estimated. Mean −log(CPO) is lower when predictive performance is better; comparisons are interpreted only within identical training samples (L1H vs NB1; NB2 vs NB3). CrI, credible interval; CS, congenital syphilis; GS, gestational syphilis; FHS, Family Health Strategy.

**Table S2. Posterior contrasts of the national temporal component across model specifications, 2015–2024.**

| **Year** | **L1H** | **NB1** | **NB2** | **NB3** |
| --- | --- | --- | --- | --- |
| 2015 | Reference | Reference | Reference | Reference |
| 2016 | 0.983 (0.909–1.052) | 0.988 (0.946–1.039) | 0.964 (0.920–1.006) | 0.981 (0.943–1.022) |
| 2017 | 0.958 (0.899–1.037) | 0.965 (0.922–1.011) | 0.865 (0.829–0.908) | 0.943 (0.909–0.985) |
| 2018 | 0.922 (0.862–0.994) | 0.930 (0.887–0.973) | 0.784 (0.744–0.821) | 0.905 (0.865–0.945) |
| 2019 | 0.875 (0.810–0.945) | 0.897 (0.859–0.941) | 0.757 (0.725–0.796) | 0.864 (0.828–0.906) |
| 2020 | 0.746 (0.687–0.808) | 0.811 (0.774–0.856) | 0.688 (0.653–0.719) | 0.792 (0.755–0.830) |
| 2021 | 0.786 (0.732–0.858) | 0.831 (0.792–0.875) | 0.695 (0.658–0.730) | 0.828 (0.791–0.867) |
| 2022 | 0.717 (0.662–0.778) | 0.782 (0.745–0.823) | 0.646 (0.614–0.679) | 0.809 (0.767–0.852) |
| 2023 | 0.675 (0.621–0.740) | 0.753 (0.713–0.796) | 0.632 (0.599–0.667) | 0.799 (0.754–0.848) |
| 2024 | 0.710 (0.654–0.781) | 0.778 (0.741–0.816) | 0.640 (0.603–0.678) | 0.816 (0.771–0.864) |

Note. Values are posterior medians (95% CrI) relative to 2015. L1H reports conditional odds-ratio contrasts; NB1–NB3 report ratios of expected CS notifications. These are contrasts of the modeled national temporal component and do not equal crude annual notification ratios.

**Table S3. Spatial sensitivity of the primary NB3 model to queen-contiguity versus symmetrized five-nearest-neighbor adjacency.**

**Panel A. Municipal associations and gestational syphilis scaling.**

| **Quantity** | **NB3 queen** | **NB3 KNN-5** | **Assessment** |
| --- | --- | --- | --- |
| Antenatal care: <7 visits, +10 percentage points | 1.097 (1.080–1.115) | 1.099 (1.081–1.117) | Stable |
| Maternal education <=7 years, +10 percentage points | 0.934 (0.915–0.952) | 0.932 (0.913–0.951) | Stable |
| Black or Brown women, +10 percentage points | 1.070 (1.056–1.085) | 1.071 (1.055–1.088) | Stable |
| FHS density, +1 team per 10,000 inhabitants | 0.889 (0.872–0.906) | 0.887 (0.871–0.905) | Stable |
| GS elasticity, delta | 0.722 (0.706–0.738) | 0.722 (0.707–0.738) | Stable |

**Panel B. Model diagnostics.**

| **Diagnostic** | **NB3 queen** | **NB3 KNN-5** |
| --- | --- | --- |
| Municipality-years | 28,436 | 28,436 |
| Municipalities | 3,625 | 3,625 |
| Mean -log(CPO) | 1.864 | 1.862 |
| Total calibration error | -0.17% | +0.35% |
| Maximum absolute annual residual Moran's I | 0.026 | 0.049 |
| Holm-significant residual Moran years | None | 2019, 2022, 2023 (negative) |
| Residual lag-1 correlation | 0.030 | 0.023 |

Note. Both models used the same 28,436 municipality-year observations from 3,625 municipalities, the same negative-binomial likelihood, freely estimated log(GS) coefficient, four prespecified covariates, covariate scaling and centering, priors, municipality-level random effect, annually replicated Besag spatial component, and first-order random-walk temporal effect; only the spatial adjacency graph was changed. Values for municipal covariates are posterior medians (95% CrI) of adjusted ratios of expected CS notifications. The GS elasticity delta is reported on the log-log scale and is not exponentiated. Mean -log(CPO) is lower when predictive performance is better and is directly comparable here because both models used the same training observations and likelihood. No positive residual spatial clustering was detected under either graph; the KNN-5 specification showed modest negative residual spatial autocorrelation in 2019, 2022, and 2023 after Holm correction. CrI, credible interval; CPO, conditional predictive ordinate; CS, congenital syphilis; GS, gestational syphilis; FHS, Family Health Strategy; KNN-5, symmetrized five-nearest-neighbor graph.

**Table S4. Sensitivity of the primary NB3 model to alternative prior specifications.**

| Quantity | Primary | More regularizing | More weakly informative |
| --- | --- | --- | --- |
| Antenatal care: <7 visits, +10 percentage points | 1.097 (1.080–1.115) | 1.097 (1.080–1.115) | 1.097 (1.080–1.115) |
| Maternal education ≤7 years, +10 percentage points | 0.934 (0.915–0.953) | 0.934 (0.915–0.952) | 0.934 (0.915–0.953) |
| Black or Brown women, +10 percentage points | 1.070 (1.056–1.085) | 1.070 (1.056–1.085) | 1.070 (1.056–1.085) |
| FHS density, +1 team per 10,000 population | 0.889 (0.872–0.906) | 0.889 (0.872–0.906) | 0.889 (0.872–0.906) |
| GS elasticity, δ | 0.722 (0.706–0.738) | 0.722 (0.706–0.738) | 0.722 (0.706–0.738) |

**Table S5. Sensitivity of the primary NB3 model to municipal maternal-age composition.**

**Panel A. Municipal associations and GS elasticity.**

| **Quantity** | **Primary NB3** | **Age-adjusted NB3** |
| --- | --- | --- |
| Antenatal visits <7, +10 percentage points | 1.097 (1.080–1.115) | 1.105 (1.087–1.123) |
| Maternal education ≤7 years, +10 percentage points | 0.934 (0.915–0.953) | 0.945 (0.925–0.964) |
| Black or Brown women, +10 percentage points | 1.070 (1.056–1.085) | 1.076 (1.061–1.091) |
| FHS teams, +1 per 10,000 inhabitants | 0.889 (0.872–0.906) | 0.893 (0.876–0.910) |
| Women aged <20 years, +10 percentage points | Not included | 0.915 (0.876–0.955) |
| Women aged ≥35 years, +10 percentage points | Not included | 1.029 (0.979–1.081) |
| GS elasticity, δ (not exponentiated) | 0.722 (0.706–0.738) | 0.720 (0.705–0.736) |

Note. Values are posterior medians (95% CrIs). Both models used the same 28,436 municipality-year observations and retained maternal education and all primary model components; only the proportions of live births to women aged <20 and ≥35 years were added. Age indicators were calculated among SINASC records with valid age and scaled per 10-percentage-point increase; ages 20–34 years form the implicit category. GS elasticity is δ=1+λ and is not exponentiated. CrI, credible interval; GS, gestational syphilis; FHS, Family Health Strategy.

**Panel B. Posterior contrasts of the national temporal component.**

| **Year** | **Primary NB3** | **Age-adjusted NB3** |
| --- | --- | --- |
| 2015 | Reference | Reference |
| 2016 | 0.981 (0.942–1.023) | 0.979 (0.941–1.019) |
| 2017 | 0.944 (0.904–0.984) | 0.932 (0.893–0.974) |
| 2018 | 0.904 (0.867–0.944) | 0.887 (0.850–0.929) |
| 2019 | 0.865 (0.828–0.908) | 0.843 (0.803–0.886) |
| 2020 | 0.793 (0.755–0.832) | 0.763 (0.724–0.803) |
| 2021 | 0.829 (0.792–0.871) | 0.797 (0.760–0.837) |
| 2022 | 0.810 (0.766–0.854) | 0.769 (0.731–0.818) |
| 2023 | 0.800 (0.760–0.845) | 0.759 (0.712–0.806) |
| 2024 | 0.816 (0.772–0.865) | 0.773 (0.730–0.825) |

Note. Values are posterior medians (95% CrIs) relative to 2015. They are contrasts of the modeled national RW1 temporal component, not crude annual notification ratios. The primary NB3 median was lowest in 2020; the age-adjusted NB3 median was lowest in 2023. CrI, credible interval; RW1, first-order random walk.

**Table S6. Fixed-design collinearity diagnostics for the primary and age-augmented NB3 models.**

| **Fixed-effect design** | **Variable** | **VIF** | **Tolerance** | **Heuristic flag** |
| --- | --- | --- | --- | --- |
| Primary | Antenatal visits <7, +10 percentage points | 1.58 | 0.635 | None |
| Primary | Maternal education ≤7 years, +10 percentage points | 1.65 | 0.607 | None |
| Primary | Black or Brown women, +10 percentage points | 1.54 | 0.648 | None |
| Primary | FHS teams, +1 per 10,000 inhabitants | 1.51 | 0.664 | None |
| Primary | Log of GS notifications (centered) | 1.33 | 0.750 | None |
| Age-augmented | Antenatal visits <7, +10 percentage points | 1.89 | 0.528 | None |
| Age-augmented | Maternal education ≤7 years, +10 percentage points | 2.10 | 0.476 | None |
| Age-augmented | Black or Brown women, +10 percentage points | 1.63 | 0.615 | None |
| Age-augmented | FHS teams, +1 per 10,000 inhabitants | 1.51 | 0.663 | None |
| Age-augmented | Log of GS notifications (centered) | 1.38 | 0.722 | None |
| Age-augmented | Women aged <20 years, +10 percentage points | 3.95 | 0.253 | None |
| Age-augmented | Women aged ≥35 years, +10 percentage points | 2.41 | 0.415 | None |

Note. Diagnostics use the identical pooled sample of 28,436 municipality-year observations and include centered log(GS). The primary design had rank 5/5 and a standardized condition index of 2.37; the age-augmented design had rank 7/7 and a condition index of 4.32. All pooled VIFs were below 5 (range, 1.33–1.65 in the primary design and 1.38–3.95 in the age-augmented design). These are diagnostics of the fixed-effect design matrix, not posterior VIFs or tests of spatial confounding. GS, gestational syphilis; VIF, variance inflation factor; FHS, Family Health Strategy.

**Table S7. Posterior predictive checks for the primary and age-adjusted NB3 models.**

| **Model** | **Statistic** | **Observed** | **Replicated 2.5th** | **Replicated median** | **Replicated 97.5th** | **Pr(rep ≥ obs)** |
| --- | --- | --- | --- | --- | --- | --- |
| Primary NB3 | Total CS notifications | 238,699 | 232,352.6 | 238,335 | 244,539.5 | 0.459 |
| Primary NB3 | Zero-count municipality-years | 8,571 | 8,148 | 8,302 | 8,462.1 | 0.000 |
| Primary NB3 | Maximum municipal-year count | 1,507 | 1,575.8 | 2,156 | 3,359.1 | 0.987 |
| Primary NB3 | 95th percentile of counts | 29 | 27 | 28 | 29 | 0.264 |
| Primary NB3 | Variance of counts | 2,090.4 | 1,856.1 | 2,219.1 | 2,702.4 | 0.711 |
| Age-adjusted NB3 | Total CS notifications | 238,699 | 232,243.4 | 238,089.5 | 244,433.3 | 0.411 |
| Age-adjusted NB3 | Zero-count municipality-years | 8,571 | 8,151 | 8,302.5 | 8,452 | 0.000 |
| Age-adjusted NB3 | Maximum municipal-year count | 1,507 | 1,574.9 | 2,160.5 | 3,378.2 | 0.986 |
| Age-adjusted NB3 | 95th percentile of counts | 29 | 27 | 28 | 29 | 0.268 |
| Age-adjusted NB3 | Variance of counts | 2,090.4 | 1,834.6 | 2,203.6 | 2,707.9 | 0.708 |

Note. Values summarize 1,000 posterior predictive replications for each model on the same 28,436 observations. Pr(rep ≥ obs) is the posterior proportion of replicated statistics at least as large as the observed statistic; it is a diagnostic tail probability, not a frequentist p-value. Both models reproduced total counts but underpredicted zero counts and overpredicted the maximum count, indicating residual distributional limitations. CS, congenital syphilis.

**Table S8. Characterization of the municipality-year panel and analytical samples.**

**Panel A. Region-year characteristics and absolute epidemiological measures.**

| **Year** | **Region** | **Municipalities in reconstructed panel** | **Municipalities with >0 live births** | **Municipalities with reported GS** | **Municipality-years included in primary NB3 model** | **Live births** | **Reported GS cases** | **Reported CS cases** | **GS detection rate per 1,000 live births** | **CS incidence rate per 1,000 live births** | **Reported CS cases per 100 reported GS cases** | **antenatal care <7 visits, median (IQR), %** | **Low maternal education, median (IQR), %** | Black or Brown women, median (IQR), % | **FHS teams per 10,000 population, median (IQR)** |
| --- | --- | --- | --- | --- | --- | --- | --- | --- | --- | --- | --- | --- | --- | --- | --- |
| 2015 | Brazil | 5,570 | 5,570 | 2,945 | 2431 | 3,017,563 | 32,799 | 19,914 | 10.87 | 6.6 | 60.72 | 27.4 (18.1–40.0) | 26.9 (18.8–36.9) | 67.7 (28.0–89.7) | 3.4 (2.5–4.2) |
| 2015 | North | 450 | 450 | 296 | 263 | 320,890 | 3,272 | 1,435 | 10.2 | 4.47 | 43.86 | 50.9 (38.2–60.7) | 35.7 (25.7–45.5) | 91.1 (80.5–95.6) | 3.1 (2.0–4.0) |
| 2015 | Northeast | 1,794 | 1,794 | 956 | 851 | 846,350 | 5,971 | 6,104 | 7.06 | 7.21 | 102.23 | 37.4 (26.4–48.8) | 37.0 (29.7–44.0) | 90.4 (82.6–95.0) | 3.8 (3.1–4.5) |
| 2015 | Midwest | 467 | 467 | 282 | 204 | 247,602 | 2,602 | 1,142 | 10.51 | 4.61 | 43.89 | 31.0 (22.8–38.5) | 20.8 (15.8–27.3) | 69.2 (57.8–78.9) | 3.2 (2.5–4.0) |
| 2015 | Southeast | 1,668 | 1,668 | 820 | 682 | 1,196,195 | 14,951 | 8,430 | 12.5 | 7.05 | 56.38 | 21.2 (15.0–28.9) | 21.8 (15.0–28.8) | 46.3 (28.6–71.3) | 3.0 (2.0–3.9) |
| 2015 | South | 1,191 | 1,191 | 591 | 431 | 406,526 | 6,003 | 2,803 | 14.77 | 6.9 | 46.69 | 19.2 (12.5–27.6) | 20.8 (15.2–28.4) | 13.0 (6.2–23.9) | 3.3 (2.3–4.1) |
| 2016 | Brazil | 5,570 | 5,570 | 3,136 | 2520 | 2,857,704 | 38,322 | 21,547 | 13.41 | 7.54 | 56.23 | 27.1 (17.9–39.0) | 24.3 (16.2–34.3) | 68.8 (28.6–90.2) | 3.4 (2.5–4.2) |
| 2016 | North | 450 | 450 | 301 | 257 | 307,495 | 3,883 | 1,755 | 12.63 | 5.71 | 45.2 | 48.1 (36.1–60.4) | 33.6 (23.3–43.3) | 90.7 (82.6–95.9) | 3.0 (2.1–4.0) |
| 2016 | Northeast | 1,794 | 1,794 | 1,000 | 883 | 796,092 | 6,602 | 6,019 | 8.29 | 7.56 | 91.17 | 35.3 (25.7–46.8) | 34.6 (27.5–41.6) | 91.2 (83.9–95.4) | 3.8 (3.1–4.5) |
| 2016 | Midwest | 467 | 467 | 279 | 213 | 234,855 | 2,901 | 1,242 | 12.35 | 5.29 | 42.81 | 29.8 (22.2–39.4) | 18.6 (13.7–25.1) | 70.1 (58.5–80.0) | 3.2 (2.5–4.0) |
| 2016 | Southeast | 1,668 | 1,668 | 898 | 716 | 1,127,473 | 18,135 | 9,368 | 16.08 | 8.31 | 51.66 | 21.2 (15.4–29.4) | 18.5 (12.2–25.9) | 47.4 (29.6–72.3) | 3.0 (2.0–3.9) |
| 2016 | South | 1,191 | 1,191 | 658 | 451 | 391,789 | 6,801 | 3,163 | 17.36 | 8.07 | 46.51 | 18.8 (12.5–27.3) | 18.5 (12.8–25.0) | 13.8 (6.7–23.8) | 3.3 (2.4–4.1) |

**Table S8. Panel A (continued): 2017–2018.**

| **Year** | **Region** | **Municipalities in reconstructed panel** | **Municipalities with >0 live births** | **Municipalities with reported GS** | **Municipality-years included in primary NB3 model** | **Live births** | **Reported GS cases** | **Reported CS cases** | **GS detection rate per 1,000 live births** | **CS incidence rate per 1,000 live births** | **Reported CS cases per 100 reported GS cases** | **antenatal care <7 visits, median (IQR), %** | **Low maternal education, median (IQR), %** | Black or Brown women, median (IQR), % | **FHS teams per 10,000 population, median (IQR)** |
| --- | --- | --- | --- | --- | --- | --- | --- | --- | --- | --- | --- | --- | --- | --- | --- |
| 2017 | Brazil | 5,570 | 5,570 | 3,563 | 2815 | 2,923,441 | 49,863 | 25,365 | 17.06 | 8.68 | 50.87 | 25.3 (16.7–36.7) | 21.6 (14.3–31.3) | 69.2 (29.9–90.3) | 3.4 (2.5–4.2) |
| 2017 | North | 450 | 450 | 352 | 294 | 312,660 | 4,760 | 2,206 | 15.22 | 7.06 | 46.34 | 46.4 (34.2–58.8) | 30.6 (21.3–40.8) | 90.7 (82.6–95.7) | 3.1 (2.2–4.0) |
| 2017 | Northeast | 1,794 | 1,794 | 1,178 | 1023 | 817,271 | 9,226 | 7,059 | 11.29 | 8.64 | 76.51 | 33.3 (24.2–44.2) | 32.0 (25.0–39.3) | 91.3 (83.8–95.5) | 3.9 (3.2–4.5) |
| 2017 | Midwest | 467 | 467 | 303 | 223 | 244,098 | 3,975 | 1,428 | 16.28 | 5.85 | 35.92 | 27.9 (21.4–36.8) | 15.6 (11.8–21.6) | 69.5 (60.3–80.0) | 3.2 (2.5–4.0) |
| 2017 | Southeast | 1,668 | 1,668 | 1,035 | 798 | 1,151,817 | 23,882 | 11,074 | 20.73 | 9.61 | 46.37 | 20.0 (14.4–27.9) | 16.3 (11.0–22.9) | 48.3 (30.9–72.7) | 3.1 (2.0–4.0) |
| 2017 | South | 1,191 | 1,191 | 695 | 477 | 397,595 | 8,020 | 3,598 | 20.17 | 9.05 | 44.86 | 17.5 (11.0–25.0) | 17.0 (12.0–23.0) | 12.9 (5.9–24.1) | 3.3 (2.4–4.1) |
| 2018 | Brazil | 5,570 | 5,570 | 3,837 | 3011 | 2,944,826 | 63,460 | 26,849 | 21.55 | 9.12 | 42.31 | 23.1 (15.4–33.3) | 19.8 (12.9–28.9) | 69.4 (30.6–90.6) | 3.5 (2.6–4.4) |
| 2018 | North | 450 | 450 | 360 | 297 | 319,199 | 5,743 | 2,249 | 17.99 | 7.05 | 39.16 | 43.8 (32.1–55.6) | 29.3 (20.0–38.0) | 91.3 (80.3–96.0) | 3.3 (2.2–4.1) |
| 2018 | Northeast | 1,794 | 1,794 | 1,319 | 1145 | 836,805 | 14,873 | 7,975 | 17.77 | 9.53 | 53.62 | 29.1 (21.3–39.0) | 29.2 (22.3–36.1) | 91.3 (84.2–95.2) | 4.0 (3.3–4.6) |
| 2018 | Midwest | 467 | 467 | 317 | 237 | 245,985 | 4,995 | 1,492 | 20.31 | 6.07 | 29.87 | 26.6 (18.6–34.8) | 14.8 (10.1–20.0) | 69.9 (61.1–79.4) | 3.3 (2.5–4.2) |
| 2018 | Southeast | 1,668 | 1,668 | 1,112 | 841 | 1,146,980 | 28,519 | 11,570 | 24.86 | 10.09 | 40.57 | 19.2 (13.9–26.2) | 14.3 (9.5–20.3) | 49.0 (31.7–72.2) | 3.2 (2.1–4.1) |
| 2018 | South | 1,191 | 1,191 | 729 | 491 | 395,857 | 9,330 | 3,563 | 23.57 | 9.0 | 38.19 | 15.6 (10.6–22.7) | 15.6 (11.1–21.4) | 14.0 (6.5–25.4) | 3.4 (2.5–4.3) |

**Table S8. Panel A (continued): 2019–2020.**

| **Year** | **Region** | **Municipalities in reconstructed panel** | **Municipalities with >0 live births** | **Municipalities with reported GS** | **Municipality-years included in primary NB3 model** | **Live births** | **Reported GS cases** | **Reported CS cases** | **GS detection rate per 1,000 live births** | **CS incidence rate per 1,000 live births** | **Reported CS cases per 100 reported GS cases** | **antenatal care <7 visits, median (IQR), %** | **Low maternal education, median (IQR), %** | Black or Brown women, median (IQR), % | **FHS teams per 10,000 population, median (IQR)** |
| --- | --- | --- | --- | --- | --- | --- | --- | --- | --- | --- | --- | --- | --- | --- | --- |
| 2019 | Brazil | 5,570 | 5,570 | 3,802 | 2962 | 2,849,064 | 64,664 | 25,394 | 22.7 | 8.91 | 39.27 | 21.2 (14.3–31.1) | 18.1 (11.5–26.5) | 70.1 (32.1–91.0) | 3.5 (2.6–4.4) |
| 2019 | North | 450 | 450 | 371 | 313 | 313,678 | 6,141 | 2,243 | 19.58 | 7.15 | 36.52 | 40.2 (29.0–53.2) | 26.2 (18.4–35.4) | 91.7 (81.6–96.0) | 3.2 (2.3–4.0) |
| 2019 | Northeast | 1,794 | 1,794 | 1,315 | 1120 | 805,252 | 14,442 | 7,097 | 17.93 | 8.81 | 49.14 | 26.1 (18.3–36.0) | 26.7 (20.8–33.9) | 91.7 (84.8–95.4) | 4.0 (3.3–4.6) |
| 2019 | Midwest | 467 | 467 | 327 | 233 | 241,077 | 5,188 | 1,425 | 21.52 | 5.91 | 27.47 | 24.1 (16.7–33.3) | 12.9 (8.7–19.2) | 70.7 (61.8–79.3) | 3.3 (2.6–4.2) |
| 2019 | Southeast | 1,668 | 1,668 | 1,075 | 818 | 1,102,964 | 29,293 | 11,339 | 26.56 | 10.28 | 38.71 | 18.1 (12.7–24.4) | 13.3 (8.4–18.6) | 50.9 (33.3–73.7) | 3.2 (2.1–4.1) |
| 2019 | South | 1,191 | 1,191 | 714 | 478 | 386,093 | 9,600 | 3,290 | 24.86 | 8.52 | 34.27 | 15.3 (10.4–21.4) | 14.1 (10.0–19.7) | 15.4 (7.8–26.3) | 3.4 (2.5–4.2) |
| 2020 | Brazil | 5,570 | 5,570 | 3,591 | 2814 | 2,730,050 | 66,168 | 23,470 | 24.24 | 8.6 | 35.47 | 22.4 (14.7–33.2) | 16.4 (10.4–24.6) | 70.9 (33.3–90.7) | 3.6 (2.8–4.5) |
| 2020 | North | 450 | 450 | 374 | 305 | 301,591 | 6,159 | 1,781 | 20.42 | 5.91 | 28.92 | 44.9 (30.0–56.7) | 24.7 (17.1–33.4) | 90.9 (81.9–95.9) | 3.5 (2.5–4.1) |
| 2020 | Northeast | 1,794 | 1,794 | 1,242 | 1052 | 770,667 | 14,125 | 6,801 | 18.33 | 8.82 | 48.15 | 28.2 (19.7–39.2) | 24.9 (18.7–31.4) | 91.7 (85.2–95.7) | 4.2 (3.4–4.7) |
| 2020 | Midwest | 467 | 467 | 315 | 227 | 230,472 | 5,314 | 1,249 | 23.06 | 5.42 | 23.5 | 26.5 (18.0–34.6) | 12.1 (8.2–16.7) | 71.9 (63.9–80.8) | 3.3 (2.6–4.2) |
| 2020 | Southeast | 1,668 | 1,668 | 994 | 765 | 1,052,375 | 31,016 | 10,538 | 29.47 | 10.01 | 33.98 | 18.5 (12.9–25.4) | 11.7 (7.5–16.7) | 51.8 (34.7–74.4) | 3.3 (2.3–4.2) |
| 2020 | South | 1,191 | 1,191 | 666 | 465 | 374,945 | 9,554 | 3,101 | 25.48 | 8.27 | 32.46 | 15.8 (10.3–23.5) | 13.2 (8.8–18.7) | 16.7 (8.6–27.2) | 3.4 (2.6–4.3) |

**Table S8. Panel A (continued): 2021–2022.**

| **Year** | **Region** | **Municipalities in reconstructed panel** | **Municipalities with >0 live births** | **Municipalities with reported GS** | **Municipality-years included in primary NB3 model** | **Live births** | **Reported GS cases** | **Reported CS cases** | **GS detection rate per 1,000 live births** | **CS incidence rate per 1,000 live births** | **Reported CS cases per 100 reported GS cases** | **antenatal care <7 visits, median (IQR), %** | **Low maternal education, median (IQR), %** | Black or Brown women, median (IQR), % | **FHS teams per 10,000 population, median (IQR)** |
| --- | --- | --- | --- | --- | --- | --- | --- | --- | --- | --- | --- | --- | --- | --- | --- |
| 2021 | Brazil | 5,570 | 5,570 | 3,867 | 2966 | 2,677,008 | 75,297 | 27,120 | 28.13 | 10.13 | 36.02 | 20.8 (14.2–30.0) | 14.9 (9.1–22.7) | 71.2 (34.7–90.6) | 3.7 (2.9–4.6) |
| 2021 | North | 450 | 450 | 375 | 316 | 309,330 | 8,060 | 2,282 | 26.06 | 7.38 | 28.31 | 38.8 (27.5–50.4) | 23.1 (15.4–30.2) | 91.4 (82.8–96.0) | 3.6 (2.8–4.3) |
| 2021 | Northeast | 1,794 | 1,794 | 1,375 | 1147 | 766,039 | 16,876 | 8,085 | 22.03 | 10.55 | 47.91 | 25.0 (17.6–34.0) | 23.0 (17.3–29.1) | 91.7 (85.2–95.6) | 4.2 (3.5–4.8) |
| 2021 | Midwest | 467 | 467 | 325 | 228 | 229,007 | 5,811 | 1,299 | 25.37 | 5.67 | 22.35 | 25.0 (17.7–34.1) | 10.0 (7.1–14.7) | 72.7 (64.2–80.8) | 3.4 (2.7–4.3) |
| 2021 | Southeast | 1,668 | 1,668 | 1,069 | 794 | 1,009,713 | 33,722 | 11,907 | 33.4 | 11.79 | 35.31 | 17.9 (12.6–24.3) | 10.4 (6.5–15.4) | 52.9 (35.7–73.6) | 3.4 (2.3–4.3) |
| 2021 | South | 1,191 | 1,191 | 723 | 481 | 362,919 | 10,828 | 3,547 | 29.84 | 9.77 | 32.76 | 15.2 (9.7–21.2) | 11.8 (7.7–16.7) | 17.8 (9.7–29.1) | 3.5 (2.7–4.4) |
| 2022 | Brazil | 5,570 | 5,570 | 4,028 | 2967 | 2,561,858 | 84,273 | 26,535 | 32.9 | 10.36 | 31.49 | 19.5 (13.2–27.2) | 13.0 (7.7–20.0) | 71.6 (35.3–90.3) | 4.0 (3.0–4.9) |
| 2022 | North | 450 | 450 | 394 | 317 | 289,149 | 8,802 | 2,387 | 30.44 | 8.26 | 27.12 | 33.5 (24.1–45.8) | 20.5 (13.9–27.6) | 90.9 (81.8–95.5) | 4.0 (3.2–4.9) |
| 2022 | Northeast | 1,794 | 1,794 | 1,343 | 1094 | 708,954 | 17,300 | 7,361 | 24.4 | 10.38 | 42.55 | 22.0 (16.0–29.5) | 19.5 (14.4–25.4) | 91.1 (84.4–95.3) | 4.6 (3.8–5.1) |
| 2022 | Midwest | 467 | 467 | 342 | 239 | 224,320 | 6,882 | 1,668 | 30.68 | 7.44 | 24.24 | 22.9 (16.7–30.4) | 9.5 (5.8–14.4) | 73.4 (65.0–81.6) | 3.6 (2.8–4.5) |
| 2022 | Southeast | 1,668 | 1,668 | 1,163 | 812 | 979,658 | 38,997 | 11,625 | 39.81 | 11.87 | 29.81 | 17.2 (12.3–23.4) | 9.1 (5.6–13.8) | 53.3 (36.3–73.8) | 3.6 (2.5–4.6) |
| 2022 | South | 1,191 | 1,191 | 786 | 505 | 359,777 | 12,292 | 3,494 | 34.17 | 9.71 | 28.42 | 14.6 (9.1–21.1) | 10.1 (6.7–14.6) | 18.5 (10.0–29.6) | 3.6 (2.8–4.5) |

**Table S8. Panel A (continued): 2023–2024.**

| **Year** | **Region** | **Municipalities in reconstructed panel** | **Municipalities with >0 live births** | **Municipalities with reported GS** | **Municipality-years included in primary NB3 model** | **Live births** | **Reported GS cases** | **Reported CS cases** | **GS detection rate per 1,000 live births** | **CS incidence rate per 1,000 live births** | **Reported CS cases per 100 reported GS cases** | **antenatal care <7 visits, median (IQR), %** | **Low maternal education, median (IQR), %** | Black or Brown women, median (IQR), % | **FHS teams per 10,000 population, median (IQR)** |
| --- | --- | --- | --- | --- | --- | --- | --- | --- | --- | --- | --- | --- | --- | --- | --- |
| 2023 | Brazil | 5,570 | 5,569 | 4,126 | 3023 | 2,537,511 | 86,545 | 25,018 | 34.11 | 9.86 | 28.91 | 17.3 (11.5–24.3) | 11.6 (6.8–18.2) | 71.2 (36.5–90.0) | 4.0 (3.1–4.9) |
| 2023 | North | 450 | 450 | 399 | 310 | 284,178 | 8,805 | 2,460 | 30.98 | 8.66 | 27.94 | 30.6 (22.1–39.5) | 18.2 (11.6–25.9) | 90.1 (81.4–94.4) | 4.1 (3.2–4.9) |
| 2023 | Northeast | 1,794 | 1,794 | 1,366 | 1127 | 703,424 | 17,445 | 6,925 | 24.8 | 9.84 | 39.7 | 19.4 (13.7–26.2) | 18.2 (13.3–23.6) | 90.6 (83.7–94.7) | 4.6 (3.9–5.1) |
| 2023 | Midwest | 467 | 467 | 361 | 244 | 226,157 | 7,822 | 1,740 | 34.59 | 7.69 | 22.24 | 20.5 (14.7–26.4) | 8.7 (5.8–13.0) | 73.3 (64.5–81.9) | 3.6 (2.8–4.6) |
| 2023 | Southeast | 1,668 | 1,667 | 1,197 | 834 | 966,143 | 40,139 | 10,572 | 41.55 | 10.94 | 26.34 | 15.6 (11.0–21.4) | 8.1 (5.0–12.2) | 54.5 (38.1–73.7) | 3.6 (2.6–4.6) |
| 2023 | South | 1,191 | 1,191 | 803 | 508 | 357,609 | 12,334 | 3,321 | 34.49 | 9.29 | 26.93 | 12.5 (8.2–18.3) | 8.6 (5.2–12.6) | 19.6 (11.1–31.0) | 3.6 (2.8–4.4) |
| 2024 | Brazil | 5,570 | 5,570 | 4,012 | 2927 | 2,389,271 | 89,719 | 24,442 | 37.55 | 10.23 | 27.24 | 15.6 (10.5–22.3) | 10.3 (5.9–16.4) | 71.4 (37.5–89.3) | 4.0 (3.1–4.9) |
| 2024 | North | 450 | 450 | 388 | 300 | 265,660 | 9,006 | 2,802 | 33.9 | 10.55 | 31.11 | 26.7 (18.8–35.5) | 16.7 (10.5–24.1) | 89.9 (80.9–94.6) | 4.2 (3.2–5.0) |
| 2024 | Northeast | 1,794 | 1,794 | 1,365 | 1085 | 667,114 | 18,243 | 7,006 | 27.35 | 10.5 | 38.4 | 17.6 (12.6–24.6) | 16.1 (11.7–21.6) | 90.2 (83.7–94.4) | 4.6 (3.9–5.2) |
| 2024 | Midwest | 467 | 467 | 331 | 226 | 215,362 | 7,824 | 1,787 | 36.33 | 8.3 | 22.84 | 17.7 (12.7–24.0) | 7.5 (5.0–11.4) | 72.7 (65.5–81.5) | 3.6 (2.8–4.6) |
| 2024 | Southeast | 1,668 | 1,668 | 1,153 | 821 | 904,335 | 42,266 | 9,720 | 46.74 | 10.75 | 23.0 | 14.2 (9.9–19.4) | 7.1 (4.1–11.1) | 54.5 (38.6–73.9) | 3.6 (2.7–4.6) |
| 2024 | South | 1,191 | 1,191 | 775 | 495 | 336,800 | 12,380 | 3,127 | 36.76 | 9.28 | 25.26 | 11.6 (7.4–16.9) | 7.3 (4.3–11.1) | 20.2 (11.4–32.9) | 3.6 (2.9–4.5) |

Note. Regional rates are ratios of summed notification counts to summed live births, not averages of municipal rates. Covariates are summarized across municipality-year observations as median (IQR). The primary NB3 sample requires at least 100 live births, at least one reported GS notification, and complete data for the four prespecified covariates; municipality-years with CS>GS are retained. Thirty GS notifications could not be allocated to the municipality of residence. The same applies to the eight unallocated CS notifications. The municipality-year panel contains 651,110 GS and 245,654 CS notifications assigned to a valid municipality of residence; Table 1 additionally includes 30 GS notifications without an allocable municipal residence code in its national descriptive total.

**Table S8. Panel B. Sequential flow into the primary NB3 analytical sample and restricted sensitivity subset.**

| **Step** | **Municipality-years retained** | **Excluded at this step** | **Municipalities** | **Reported GS cases retained** | **Reported CS cases retained** | **Live births retained** | **Retained from previous step (%)** |
| --- | --- | --- | --- | --- | --- | --- | --- |
| Complete reconstructed panel | 55700 | 0 | 5570 | 651110 | 245654 | 27488296 | 100.0 |
| Positive live-birth denominator | 55699 | 1 | 5570 | 651110 | 245654 | 27488296 | 100.0 |
| At least 100 live births | 33588 | 22111 | 3717 | 636849 | 240376 | 26309781 | 60.3 |
| At least 1 reported GS notification | 28436 | 5152 | 3625 | 636849 | 238699 | 25305992 | 84.7 |
| Complete data for four prespecified covariates (primary NB3 sample) | 28436 | 0 | 3625 | 636849 | 238699 | 25305992 | 100.0 |
| Restricted sensitivity subset: reported CS not exceeding reported GS | 26513 | 1923 | 3622 | 619408 | 210245 | 23514792 | 93.2 |

Note. The CS≤GS restriction is not part of the primary NB3 sample. It defines the restricted sample used for the harmonized binomial model (L1H) and NB1 sensitivity analysis. Table 1 is a notification-level national descriptive table; the present table summarizes the reconstructed municipality-year panel used in the ecological analyses.”.

**Table S8. Panel C. Missingness of the four prespecified covariates in the primary-model eligible sample.**

| **Year** | **Variable** | **Eligible municipality-years before complete-case restriction** | **Missing municipality-years** | **Missing (%)** |
| --- | --- | --- | --- | --- |
| Overall | antenatal care <7 visits | 28436 | 0 | 0.0 |
| Overall | Low maternal education (<=7 years) | 28436 | 0 | 0.0 |
| Overall | Black or Brown woman | 28436 | 0 | 0.0 |
| Overall | FHS teams per 10,000 population | 28436 | 0 | 0.0 |
| 2015 | antenatal care <7 visits | 2431 | 0 | 0.0 |
| 2015 | Low maternal education (<=7 years) | 2431 | 0 | 0.0 |
| 2015 | Black or Brown woman | 2431 | 0 | 0.0 |
| 2015 | FHS teams per 10,000 population | 2431 | 0 | 0.0 |
| 2016 | antenatal care <7 visits | 2520 | 0 | 0.0 |
| 2016 | Low maternal education (<=7 years) | 2520 | 0 | 0.0 |
| 2016 | Black or Brown woman | 2520 | 0 | 0.0 |
| 2016 | FHS teams per 10,000 population | 2520 | 0 | 0.0 |
| 2017 | antenatal care <7 visits | 2815 | 0 | 0.0 |
| 2017 | Low maternal education (<=7 years) | 2815 | 0 | 0.0 |
| 2017 | Black or Brown woman | 2815 | 0 | 0.0 |
| 2017 | FHS teams per 10,000 population | 2815 | 0 | 0.0 |

**Table S8. Panel C (continued): 2018–2021.**

| **Year** | **Variable** | **Eligible municipality-years before complete-case restriction** | **Missing municipality-years** | **Missing (%)** |
| --- | --- | --- | --- | --- |
| 2018 | antenatal care <7 visits | 3011 | 0 | 0.0 |
| 2018 | Low maternal education (<=7 years) | 3011 | 0 | 0.0 |
| 2018 | Black or Brown woman | 3011 | 0 | 0.0 |
| 2018 | FHS teams per 10,000 population | 3011 | 0 | 0.0 |
| 2019 | antenatal care <7 visits | 2962 | 0 | 0.0 |
| 2019 | Low maternal education (<=7 years) | 2962 | 0 | 0.0 |
| 2019 | Black or Brown woman | 2962 | 0 | 0.0 |
| 2019 | FHS teams per 10,000 population | 2962 | 0 | 0.0 |
| 2020 | antenatal care <7 visits | 2814 | 0 | 0.0 |
| 2020 | Low maternal education (<=7 years) | 2814 | 0 | 0.0 |
| 2020 | Black or Brown woman | 2814 | 0 | 0.0 |
| 2020 | FHS teams per 10,000 population | 2814 | 0 | 0.0 |
| 2021 | antenatal care <7 visits | 2966 | 0 | 0.0 |
| 2021 | Low maternal education (<=7 years) | 2966 | 0 | 0.0 |
| 2021 | Black or Brown woman | 2966 | 0 | 0.0 |
| 2021 | FHS teams per 10,000 population | 2966 | 0 | 0.0 |

**Table S8. Panel C (continued): 2022–2024.**

| **Year** | **Variable** | **Eligible municipality-years before complete-case restriction** | **Missing municipality-years** | **Missing (%)** |
| --- | --- | --- | --- | --- |
| 2022 | antenatal care <7 visits | 2967 | 0 | 0.0 |
| 2022 | Low maternal education (<=7 years) | 2967 | 0 | 0.0 |
| 2022 | Black or Brown woman | 2967 | 0 | 0.0 |
| 2022 | FHS teams per 10,000 population | 2967 | 0 | 0.0 |
| 2023 | antenatal care <7 visits | 3023 | 0 | 0.0 |
| 2023 | Low maternal education (<=7 years) | 3023 | 0 | 0.0 |
| 2023 | Black or Brown woman | 3023 | 0 | 0.0 |
| 2023 | FHS teams per 10,000 population | 3023 | 0 | 0.0 |
| 2024 | antenatal care <7 visits | 2927 | 0 | 0.0 |
| 2024 | Low maternal education (<=7 years) | 2927 | 0 | 0.0 |
| 2024 | Black or Brown woman | 2927 | 0 | 0.0 |
| 2024 | FHS teams per 10,000 population | 2927 | 0 | 0.0 |

Note. No municipality-year was excluded for missing values in the four prespecified covariates after the live-birth and GS-notification eligibility criteria were applied.

**Table S8. Panel D. Distribution of municipalities by number of years contributed to the primary NB3 model.**

| **Region** | **0** | **1** | **2** | **3** | **4** | **5** | **6** | **7** | **8** | **9** | **10** |
| --- | --- | --- | --- | --- | --- | --- | --- | --- | --- | --- | --- |
| Brazil | 1945 (34.9%) | 159 (2.9%) | 134 (2.4%) | 121 (2.2%) | 147 (2.6%) | 155 (2.8%) | 190 (3.4%) | 255 (4.6%) | 383 (6.9%) | 516 (9.3%) | 1565 (28.1%) |
| Midwest | 180 (38.5%) | 14 (3.0%) | 16 (3.4%) | 10 (2.1%) | 11 (2.4%) | 9 (1.9%) | 5 (1.1%) | 18 (3.9%) | 27 (5.8%) | 33 (7.1%) | 144 (30.8%) |
| North | 104 (23.1%) | 8 (1.8%) | 5 (1.1%) | 6 (1.3%) | 11 (2.4%) | 7 (1.6%) | 13 (2.9%) | 16 (3.6%) | 39 (8.7%) | 55 (12.2%) | 186 (41.3%) |
| Northeast | 409 (22.8%) | 58 (3.2%) | 53 (3.0%) | 50 (2.8%) | 65 (3.6%) | 73 (4.1%) | 89 (5.0%) | 114 (6.4%) | 167 (9.3%) | 240 (13.4%) | 476 (26.5%) |
| South | 599 (50.3%) | 25 (2.1%) | 18 (1.5%) | 21 (1.8%) | 23 (1.9%) | 20 (1.7%) | 28 (2.4%) | 36 (3.0%) | 50 (4.2%) | 64 (5.4%) | 307 (25.8%) |
| Southeast | 653 (39.1%) | 54 (3.2%) | 42 (2.5%) | 34 (2.0%) | 37 (2.2%) | 46 (2.8%) | 55 (3.3%) | 71 (4.3%) | 100 (6.0%) | 124 (7.4%) | 452 (27.1%) |

Note. The primary NB3 model included 28,436 municipality-year observations from 3,625 municipalities. Municipalities with zero contributed years remain represented in the nationwide descriptive panel but not in the primary inferential model.

**Table S8. Panel E. SINASC source-record completeness in the primary NB3 sample, by year and region.**

| **Year** | **Region** | **Municipality-years** | **SINASC records** | **Age nonvalid (%)** | **Education nonvalid (%)** | **Race/skin color nonvalid (%)** | **Antenatal visits nonvalid (%)** |
| --- | --- | --- | --- | --- | --- | --- | --- |
| 2015 | Brazil | 2,431 | 2,680,863 | 0.004 | 1.36 | 4.93 | 0.62 |
| 2015 | North | 263 | 295,150 | 0.017 | 1.88 | 3.77 | 0.44 |
| 2015 | Northeast | 851 | 702,749 | 0.003 | 2.36 | 8.04 | 0.74 |
| 2015 | Southeast | 682 | 1,101,780 | 0.002 | 1.00 | 2.99 | 0.71 |
| 2015 | South | 431 | 355,584 | 0.000 | 0.28 | 1.74 | 0.22 |
| 2015 | Midwest | 204 | 225,600 | 0.007 | 1.07 | 11.24 | 0.64 |
| 2016 | Brazil | 2,520 | 2,553,249 | 0.002 | 1.26 | 4.53 | 0.61 |
| 2016 | North | 257 | 278,465 | 0.006 | 1.78 | 3.64 | 0.47 |
| 2016 | Northeast | 883 | 667,797 | 0.002 | 2.29 | 7.51 | 0.69 |
| 2016 | Southeast | 716 | 1,045,635 | 0.002 | 0.90 | 2.45 | 0.72 |
| 2016 | South | 451 | 344,683 | 0.002 | 0.28 | 1.47 | 0.31 |
| 2016 | Midwest | 213 | 216,669 | 0.002 | 0.74 | 11.43 | 0.47 |
| 2017 | Brazil | 2,815 | 2,688,723 | 0.002 | 1.37 | 4.17 | 0.48 |
| 2017 | North | 294 | 293,647 | 0.006 | 2.37 | 2.86 | 0.27 |
| 2017 | Northeast | 1,023 | 727,792 | 0.001 | 2.73 | 8.11 | 0.61 |
| 2017 | Southeast | 798 | 1,084,973 | 0.001 | 0.68 | 1.48 | 0.57 |
| 2017 | South | 477 | 355,034 | 0.001 | 0.29 | 1.41 | 0.18 |
| 2017 | Midwest | 223 | 227,277 | 0.003 | 0.71 | 10.41 | 0.37 |
| 2018 | Brazil | 3,011 | 2,751,942 | 0.003 | 1.45 | 3.58 | 0.44 |
| 2018 | North | 297 | 302,264 | 0.015 | 2.68 | 2.09 | 0.26 |
| 2018 | Northeast | 1,145 | 774,095 | 0.001 | 3.01 | 7.02 | 0.45 |
| 2018 | Southeast | 841 | 1,088,440 | 0.001 | 0.50 | 1.12 | 0.53 |
| 2018 | South | 491 | 355,902 | 0.001 | 0.42 | 1.31 | 0.23 |
| 2018 | Midwest | 237 | 231,241 | 0.003 | 0.64 | 9.09 | 0.52 |
| 2019 | Brazil | 2,962 | 2,649,118 | 0.003 | 1.15 | 3.43 | 0.45 |
| 2019 | North | 313 | 302,207 | 0.012 | 1.91 | 2.70 | 0.28 |
| 2019 | Northeast | 1,120 | 738,258 | 0.001 | 2.31 | 7.15 | 0.53 |
| 2019 | Southeast | 818 | 1,039,515 | 0.003 | 0.54 | 1.13 | 0.48 |
| 2019 | South | 478 | 342,743 | 0.002 | 0.29 | 0.98 | 0.26 |
| 2019 | Midwest | 233 | 226,395 | 0.003 | 0.46 | 6.52 | 0.56 |
| 2020 | Brazil | 2,814 | 2,509,722 | 0.003 | 1.06 | 3.30 | 0.53 |
| 2020 | North | 305 | 289,515 | 0.012 | 1.61 | 2.12 | 0.63 |
| 2020 | Northeast | 1,052 | 691,369 | 0.002 | 2.02 | 7.45 | 0.68 |
| 2020 | Southeast | 765 | 981,693 | 0.001 | 0.59 | 1.26 | 0.49 |
| 2020 | South | 465 | 332,429 | 0.000 | 0.32 | 0.89 | 0.26 |
| 2020 | Midwest | 227 | 214,716 | 0.001 | 0.55 | 4.63 | 0.53 |
| 2021 | Brazil | 2,966 | 2,489,497 | 0.003 | 1.15 | 3.34 | 0.54 |
| 2021 | North | 316 | 299,680 | 0.016 | 1.93 | 2.22 | 0.41 |
| 2021 | Northeast | 1,147 | 705,811 | 0.001 | 2.01 | 7.44 | 0.82 |
| 2021 | Southeast | 794 | 945,798 | 0.001 | 0.60 | 1.21 | 0.49 |
| 2021 | South | 481 | 324,670 | 0.001 | 0.68 | 1.19 | 0.30 |
| 2021 | Midwest | 228 | 213,538 | 0.003 | 0.34 | 4.08 | 0.43 |
| 2022 | Brazil | 2,967 | 2,386,695 | 0.002 | 0.89 | 2.53 | 0.62 |
| 2022 | North | 317 | 279,441 | 0.009 | 2.24 | 1.74 | 0.55 |
| 2022 | Northeast | 1,094 | 646,958 | 0.001 | 1.42 | 5.42 | 1.11 |
| 2022 | Southeast | 812 | 924,086 | 0.002 | 0.45 | 1.19 | 0.54 |
| 2022 | South | 505 | 324,619 | 0.001 | 0.31 | 1.13 | 0.19 |
| 2022 | Midwest | 239 | 211,591 | 0.000 | 0.31 | 2.76 | 0.21 |
| 2023 | Brazil | 3,023 | 2,372,679 | 0.002 | 0.65 | 2.05 | 0.43 |
| 2023 | North | 310 | 273,701 | 0.008 | 1.08 | 1.61 | 0.90 |
| 2023 | Northeast | 1,127 | 648,745 | 0.001 | 1.23 | 4.69 | 0.74 |
| 2023 | Southeast | 834 | 914,639 | 0.001 | 0.32 | 0.89 | 0.24 |
| 2023 | South | 508 | 322,379 | 0.000 | 0.34 | 1.01 | 0.14 |
| 2023 | Midwest | 244 | 213,215 | 0.002 | 0.24 | 1.11 | 0.14 |
| 2024 | Brazil | 2,927 | 2,223,504 | 0.002 | 0.56 | 1.88 | 0.43 |
| 2024 | North | 300 | 254,968 | 0.009 | 0.97 | 1.72 | 1.37 |
| 2024 | Northeast | 1,085 | 608,925 | 0.002 | 0.98 | 3.88 | 0.51 |
| 2024 | Southeast | 821 | 855,022 | 0.001 | 0.29 | 1.21 | 0.26 |
| 2024 | South | 495 | 303,073 | 0.000 | 0.34 | 0.87 | 0.13 |
| 2024 | Midwest | 226 | 201,516 | 0.000 | 0.21 | 0.43 | 0.18 |

Note. Percentages are the proportions of individual SINASC live-birth records with missing, unknown, or invalid information for each field among the 25,305,992 source records contributing to the 28,436 municipality-year observations in the primary NB3 sample. Across all years and regions combined, nonvalid information was 0.003% for age, 1.11% for education, 3.42% for race/skin color, and 0.52% for antenatal visits. All municipality-year aggregates remained estimable using field-specific valid-record denominators; no imputation was performed. SINASC, Live Birth Information System.

**Table S9. Annual Global Moran's I for empirical-Bayes-smoothed gestational and congenital syphilis rates, Brazil, 2015–2024.**

| **Year** | **Gestational syphilis Moran's I** | **Gestational syphilis p-value** | **Congenital syphilis Moran's I** | **Congenital syphilis p-value** |
| --- | --- | --- | --- | --- |
| 2015 | 0.223 | 0.001 | 0.306 | 0.001 |
| 2016 | 0.25 | 0.001 | 0.299 | 0.001 |
| 2017 | 0.236 | 0.001 | 0.304 | 0.001 |
| 2018 | 0.272 | 0.001 | 0.291 | 0.001 |
| 2019 | 0.272 | 0.001 | 0.339 | 0.001 |
| 2020 | 0.275 | 0.001 | 0.348 | 0.001 |
| 2021 | 0.301 | 0.001 | 0.357 | 0.001 |
| 2022 | 0.297 | 0.001 | 0.324 | 0.001 |
| 2023 | 0.289 | 0.001 | 0.293 | 0.001 |
| 2024 | 0.308 | 0.001 | 0.3 | 0.001 |

Note. First-order queen contiguity and row-standardized spatial weights were used; p-values are based on 999 Monte Carlo permutations. Spatial analyses are descriptive and are independent of the inferential NB3 model.

**Table S10. Distribution of LISA cluster types for congenital syphilis across Brazilian regions, aggregated over municipality-year observations, 2015–2024.**

| **Region** | **Total municipality-years** | **High-High** | **Low-Low** | **High-Low** | **Low-High** | **Not significant** | **Missing** |
| --- | --- | --- | --- | --- | --- | --- | --- |
| North | 4500 | 372 (8.3%) | 530 (11.8%) | 62 (1.4%) | 160 (3.6%) | 3376 (75.0%) | 0 (0.0%) |
| Northeast | 17940 | 1328 (7.4%) | 941 (5.2%) | 79 (0.4%) | 360 (2.0%) | 15232 (84.9%) | 0 (0.0%) |
| Midwest | 4670 | 108 (2.3%) | 84 (1.8%) | 19 (0.4%) | 67 (1.4%) | 4392 (94.0%) | 0 (0.0%) |
| Southeast | 16680 | 993 (6.0%) | 388 (2.3%) | 45 (0.3%) | 460 (2.8%) | 14793 (88.7%) | 1 (0.0%) |
| South | 11910 | 642 (5.4%) | 148 (1.2%) | 11 (0.1%) | 255 (2.1%) | 10854 (91.1%) | 0 (0.0%) |

Note. Percentages use all municipality-year observations within each region. Missing is shown explicitly. Local LISA classification used the empirical-Bayes-smoothed congenital syphilis rate and queen contiguity.

**Table S11. Distribution of LISA cluster types for congenital syphilis across Brazilian federative units, aggregated over municipality-year observations, 2015–2024.**

| **State** | **Total municipality-years** | **High-High** | **Low-Low** | **High-Low** | **Low-High** | **Not significant** | **Missing** |
| --- | --- | --- | --- | --- | --- | --- | --- |
| AC | 220 | 6 (2.7%) | 13 (5.9%) | 6 (2.7%) | 4 (1.8%) | 191 (86.8%) | 0 (0.0%) |
| AL | 1020 | 90 (8.8%) | 124 (12.2%) | 4 (0.4%) | 25 (2.5%) | 777 (76.2%) | 0 (0.0%) |
| AM | 620 | 1 (0.2%) | 258 (41.6%) | 21 (3.4%) | 0 (0.0%) | 340 (54.8%) | 0 (0.0%) |
| AP | 160 | 2 (1.2%) | 15 (9.4%) | 1 (0.6%) | 0 (0.0%) | 142 (88.8%) | 0 (0.0%) |
| BA | 4170 | 72 (1.7%) | 256 (6.1%) | 22 (0.5%) | 31 (0.7%) | 3789 (90.9%) | 0 (0.0%) |
| CE | 1840 | 69 (3.8%) | 73 (4.0%) | 8 (0.4%) | 24 (1.3%) | 1666 (90.5%) | 0 (0.0%) |
| DF | 10 | 4 (40.0%) | 0 (0.0%) | 0 (0.0%) | 2 (20.0%) | 4 (40.0%) | 0 (0.0%) |
| ES | 780 | 89 (11.4%) | 11 (1.4%) | 5 (0.6%) | 32 (4.1%) | 643 (82.4%) | 0 (0.0%) |
| GO | 2460 | 57 (2.3%) | 3 (0.1%) | 3 (0.1%) | 26 (1.1%) | 2371 (96.4%) | 0 (0.0%) |
| MA | 2170 | 69 (3.2%) | 416 (19.2%) | 29 (1.3%) | 16 (0.7%) | 1640 (75.6%) | 0 (0.0%) |
| MG | 8530 | 385 (4.5%) | 44 (0.5%) | 8 (0.1%) | 202 (2.4%) | 7891 (92.5%) | 0 (0.0%) |
| MS | 790 | 45 (5.7%) | 3 (0.4%) | 2 (0.3%) | 36 (4.6%) | 704 (89.1%) | 0 (0.0%) |
| MT | 1410 | 2 (0.1%) | 78 (5.5%) | 14 (1.0%) | 3 (0.2%) | 1313 (93.1%) | 0 (0.0%) |
| PA | 1440 | 66 (4.6%) | 132 (9.2%) | 21 (1.5%) | 39 (2.7%) | 1182 (82.1%) | 0 (0.0%) |
| PB | 2230 | 24 (1.1%) | 13 (0.6%) | 1 (0.0%) | 15 (0.7%) | 2177 (97.6%) | 0 (0.0%) |
| PE | 1850 | 418 (22.6%) | 48 (2.6%) | 10 (0.5%) | 65 (3.5%) | 1309 (70.8%) | 0 (0.0%) |
| PI | 2240 | 106 (4.7%) | 8 (0.4%) | 2 (0.1%) | 45 (2.0%) | 2079 (92.8%) | 0 (0.0%) |
| PR | 3990 | 83 (2.1%) | 108 (2.7%) | 6 (0.2%) | 38 (1.0%) | 3755 (94.1%) | 0 (0.0%) |
| RJ | 920 | 268 (29.1%) | 8 (0.9%) | 0 (0.0%) | 96 (10.4%) | 548 (59.6%) | 0 (0.0%) |
| RN | 1670 | 237 (14.2%) | 0 (0.0%) | 0 (0.0%) | 77 (4.6%) | 1356 (81.2%) | 0 (0.0%) |
| RO | 520 | 0 (0.0%) | 104 (20.0%) | 9 (1.7%) | 0 (0.0%) | 407 (78.3%) | 0 (0.0%) |
| RR | 150 | 1 (0.7%) | 8 (5.3%) | 4 (2.7%) | 5 (3.3%) | 132 (88.0%) | 0 (0.0%) |
| RS | 4970 | 424 (8.5%) | 0 (0.0%) | 0 (0.0%) | 168 (3.4%) | 4378 (88.1%) | 0 (0.0%) |
| SC | 2950 | 135 (4.6%) | 40 (1.4%) | 5 (0.2%) | 49 (1.7%) | 2721 (92.2%) | 0 (0.0%) |
| SE | 750 | 243 (32.4%) | 3 (0.4%) | 3 (0.4%) | 62 (8.3%) | 439 (58.5%) | 0 (0.0%) |
| SP | 6450 | 251 (3.9%) | 325 (5.0%) | 32 (0.5%) | 130 (2.0%) | 5711 (88.5%) | 1 (0.0%) |
| TO | 1390 | 296 (21.3%) | 0 (0.0%) | 0 (0.0%) | 112 (8.1%) | 982 (70.6%) | 0 (0.0%) |

Note. Percentages use all municipality-year observations within each state. Missing is shown explicitly.

**Table S12. Distribution of LISA cluster types for gestational syphilis across Brazilian regions, aggregated over municipality-year observations, 2015–2024.**

| **Region** | **Total municipality-years** | **High-High** | **Low-Low** | **High-Low** | **Low-High** | **Not significant** | **Missing** |
| --- | --- | --- | --- | --- | --- | --- | --- |
| North | 4500 | 343 (7.6%) | 308 (6.8%) | 65 (1.4%) | 106 (2.4%) | 3678 (81.7%) | 0 (0.0%) |
| Northeast | 17940 | 525 (2.9%) | 1892 (10.5%) | 271 (1.5%) | 191 (1.1%) | 15061 (84.0%) | 0 (0.0%) |
| Midwest | 4670 | 427 (9.1%) | 24 (0.5%) | 19 (0.4%) | 171 (3.7%) | 4029 (86.3%) | 0 (0.0%) |
| Southeast | 16680 | 875 (5.2%) | 403 (2.4%) | 83 (0.5%) | 356 (2.1%) | 14962 (89.7%) | 1 (0.0%) |
| South | 11910 | 1189 (10.0%) | 34 (0.3%) | 7 (0.1%) | 513 (4.3%) | 10167 (85.4%) | 0 (0.0%) |

Note. Percentages use all municipality-year observations within each region. Missing is shown explicitly. Local LISA classification used the empirical-Bayes-smoothed gestational syphilis detection rate and queen contiguity.

**Table S13. Distribution of gestational syphilis LISA cluster types by Brazilian federative unit, 2015–2024.**

| **State** | **Total municipality-years** | **High-High** | **Low-Low** | **High-Low** | **Low-High** | **Not significant** | **Missing** |
| --- | --- | --- | --- | --- | --- | --- | --- |
| AC | 220 | 73 (33.2%) | 0 (0.0%) | 3 (1.4%) | 12 (5.5%) | 132 (60.0%) | 0 (0.0%) |
| AL | 1020 | 8 (0.8%) | 182 (17.8%) | 14 (1.4%) | 5 (0.5%) | 811 (79.5%) | 0 (0.0%) |
| AM | 620 | 2 (0.3%) | 168 (27.1%) | 19 (3.1%) | 5 (0.8%) | 426 (68.7%) | 0 (0.0%) |
| AP | 160 | 0 (0.0%) | 3 (1.9%) | 2 (1.2%) | 1 (0.6%) | 154 (96.2%) | 0 (0.0%) |
| BA | 4170 | 193 (4.6%) | 505 (12.1%) | 52 (1.2%) | 97 (2.3%) | 3323 (79.7%) | 0 (0.0%) |
| CE | 1840 | 5 (0.3%) | 202 (11.0%) | 42 (2.3%) | 5 (0.3%) | 1586 (86.2%) | 0 (0.0%) |
| DF | 10 | 4 (40.0%) | 0 (0.0%) | 0 (0.0%) | 1 (10.0%) | 5 (50.0%) | 0 (0.0%) |
| ES | 780 | 89 (11.4%) | 100 (12.8%) | 15 (1.9%) | 12 (1.5%) | 564 (72.3%) | 0 (0.0%) |
| GO | 2460 | 130 (5.3%) | 9 (0.4%) | 5 (0.2%) | 60 (2.4%) | 2256 (91.7%) | 0 (0.0%) |
| MA | 2170 | 17 (0.8%) | 586 (27.0%) | 85 (3.9%) | 8 (0.4%) | 1474 (67.9%) | 0 (0.0%) |
| MG | 8530 | 175 (2.1%) | 138 (1.6%) | 29 (0.3%) | 103 (1.2%) | 8085 (94.8%) | 0 (0.0%) |
| MS | 790 | 266 (33.7%) | 0 (0.0%) | 1 (0.1%) | 94 (11.9%) | 429 (54.3%) | 0 (0.0%) |
| MT | 1410 | 27 (1.9%) | 15 (1.1%) | 13 (0.9%) | 16 (1.1%) | 1339 (95.0%) | 0 (0.0%) |
| PA | 1440 | 65 (4.5%) | 102 (7.1%) | 30 (2.1%) | 27 (1.9%) | 1216 (84.4%) | 0 (0.0%) |
| PB | 2230 | 2 (0.1%) | 121 (5.4%) | 15 (0.7%) | 1 (0.0%) | 2091 (93.8%) | 0 (0.0%) |
| PE | 1850 | 112 (6.1%) | 172 (9.3%) | 36 (1.9%) | 16 (0.9%) | 1514 (81.8%) | 0 (0.0%) |
| PI | 2240 | 1 (0.0%) | 99 (4.4%) | 15 (0.7%) | 6 (0.3%) | 2119 (94.6%) | 0 (0.0%) |
| PR | 3990 | 218 (5.5%) | 19 (0.5%) | 6 (0.2%) | 128 (3.2%) | 3619 (90.7%) | 0 (0.0%) |
| RJ | 920 | 314 (34.1%) | 10 (1.1%) | 1 (0.1%) | 85 (9.2%) | 510 (55.4%) | 0 (0.0%) |
| RN | 1670 | 59 (3.5%) | 19 (1.1%) | 2 (0.1%) | 19 (1.1%) | 1571 (94.1%) | 0 (0.0%) |
| RO | 520 | 25 (4.8%) | 27 (5.2%) | 7 (1.3%) | 5 (1.0%) | 456 (87.7%) | 0 (0.0%) |
| RR | 150 | 3 (2.0%) | 7 (4.7%) | 4 (2.7%) | 4 (2.7%) | 132 (88.0%) | 0 (0.0%) |
| RS | 4970 | 742 (14.9%) | 0 (0.0%) | 1 (0.0%) | 270 (5.4%) | 3957 (79.6%) | 0 (0.0%) |
| SC | 2950 | 229 (7.8%) | 15 (0.5%) | 0 (0.0%) | 115 (3.9%) | 2591 (87.8%) | 0 (0.0%) |
| SE | 750 | 128 (17.1%) | 6 (0.8%) | 10 (1.3%) | 34 (4.5%) | 572 (76.3%) | 0 (0.0%) |
| SP | 6450 | 297 (4.6%) | 155 (2.4%) | 38 (0.6%) | 156 (2.4%) | 5803 (90.0%) | 1 (0.0%) |
| TO | 1390 | 175 (12.6%) | 1 (0.1%) | 0 (0.0%) | 52 (3.7%) | 1162 (83.6%) | 0 (0.0%) |

Note. Percentages use all municipality-year observations within each state. Missing is shown explicitly.
