# Supplementary material for: Congenital Syphilis in Brazil: Municipal Antenatal Care, Primary Health Care Capacity, and Territorial Inequalities, 2015–2024
Source: Matern Child Health J. 2026 Sep 19;30(10):1304–13. doi: 10.1007/s10995-026-04329-1 (PMC13672521; doi:10.1007/s10995-026-04329-1)
Supplement: Supplementary file 2 [file 10995_2026_4329_MOESM12_ESM.docx]

**Online Resource 1. Completed RECORD/STROBE and SAMPL Reporting Checklists**

*Manuscript: Congenital syphilis in Brazil: municipal antenatal care, primary health care capacity, and territorial inequalities, 2015–2024*

**A. RECORD/STROBE checklist**

| **Item** | **Reporting recommendation** | **Where reported in manuscript** |
| --- | --- | --- |
| STROBE 1a | Indicate the study design with a commonly used term in the title or abstract. | p. 1, lines 1–4 |
| STROBE 1b | Provide an informative and balanced summary of what was done and what was found. | p. 1, lines 5–29 |
| RECORD 1.1 | State in the abstract the type of routinely collected data used. | p. 1, lines 9–14 |
| RECORD 1.2 | Where possible, include the geographic region and timeframe in the title or abstract. | p. 1, lines 1–14 |
| RECORD 1.3 | If linkage of routinely collected data was performed, state this in the title or abstract when appropriate. | N/A (no individual record linkage) |
| STROBE 2 | Explain the scientific background and rationale for the investigation. | p. 3, lines 44–73 |
| STROBE 3 | State specific objectives, including any prespecified hypotheses. | pp. 3–4, lines 74–76 |
| STROBE 4 | Present key elements of the study design early in the paper. | p. 4, lines 79–97 |
| STROBE 5 | Describe setting, locations and relevant dates, including data-collection period. | p. 4, lines 79–97 |
| STROBE 6 | Give eligibility criteria and the sources/methods of selection of study units. | p. 4, lines 79–97; p. 6, lines 135–139; p. 8, lines 206–212; Online Resource 2, Table S8 |
| RECORD 6.1 | Describe in detail the selection of the study population from the routinely collected data, including codes/algorithms where applicable. | p. 4, lines 89–97; p. 6, lines 135–139; Online Resource 3 (supplementary code) |
| RECORD 6.2 | Describe validation of codes/algorithms used to identify the study population, or explain why validation was not applicable. | N/A |
| RECORD 6.3 | If linkage was performed, report a linkage flow diagram or sufficient detail on linkage success. | N/A (no individual record linkage) |
| STROBE 7 | Clearly define outcomes, exposures, predictors, potential confounders and effect modifiers. | pp. 4–5, lines 99–123 |
| RECORD 7.1 | Provide a complete list of codes/algorithms used to classify exposures, outcomes, confounders and effect modifiers. | p. 5, lines 108–123; Online Resource 3 (supplementary code) |
| STROBE 8 | For each variable, give sources of data and details of assessment/measurement. | pp. 4–5, lines 79–123 |
| STROBE 9 | Describe efforts to address potential sources of bias. | p. 4, lines 89–97; p. 6, lines 135–163; pp. 7–8, lines 165–202; p. 12, lines 329–348 |
| STROBE 10 | Explain how the study size was arrived at. | p. 6, lines 135–139; p. 8, lines 206–212; Online Resource 2, Table S8 |
| STROBE 11 | Explain how quantitative variables were handled and justify groupings/cut-points when relevant. | p. 5, lines 108–123 |
| STROBE 12a | Describe all statistical methods, including methods used to control for confounding. | pp. 4–8, lines 99–202 |
| STROBE 12b | Describe methods used to examine subgroups and interactions. | N/A |
| STROBE 12c | Explain how missing data were addressed. | p. 8, lines 195–212; p. 12, lines 329–344; Online Resource 2, Table S8, Panels C and E |
| STROBE 12d | If applicable, explain how loss to follow-up, sampling strategy or matching was handled. | N/A |
| STROBE 12e | Describe sensitivity analyses. | p. 7, lines 165–194; Online Resource 2, Tables S1–S7 |
| RECORD 12.1 | Describe the extent to which investigators had access to the database population used to create the study population. | p. 8, lines 195–202; Online Resource 3 (supplementary code) |
| RECORD 12.2 | Describe data-cleaning methods. | p. 4, lines 89–97; p. 8, lines 195–202; Online Resource 3 (supplementary code) |
| RECORD 12.3 | If linkage was performed, state the linkage level and method, and assess linkage quality. | N/A (no pregnancy–infant record linkage; pp. 4–5, lines 103–107) |
| STROBE 13 | Report numbers of study units at each stage and reasons for non-participation/exclusion; consider a flow diagram. | p. 6, lines 135–139; p. 8, lines 206–212; Online Resource 2, Table S8, Panel B |
| RECORD 13.1 | Describe selection of the study population using information from the routinely collected data. | p. 6, lines 135–139; p. 8, lines 206–212; Online Resource 2, Table S8, Panel B |
| STROBE 14 | Give descriptive characteristics and information on missing data. | p. 8, lines 206–224; Table 1, p. 16; Online Resource 2, Table S8, Panels A, C, and E |
| STROBE 15 | Report outcome events or summary measures over time/strata as appropriate. | pp. 8–9, lines 213–236; Online Resource 2, Tables S8–S13 |
| STROBE 16a | Give unadjusted and adjusted estimates with precision; state confounders and why they were included. | p. 9, lines 238–249; Table 2, p. 19, lines 466–478 |
| STROBE 16b | Report category boundaries when continuous variables are categorized. | p. 5, lines 108–123; Table 2, p. 19, lines 466–478 |
| STROBE 16c | Translate relative risk estimates into absolute risk for a meaningful time period when relevant. | N/A |
| STROBE 17 | Report other analyses, including sensitivity analyses. | pp. 9–10, lines 250–262; Online Resource 2, Tables S1–S7 |
| **DISCUSSION AND OTHER INFORMATION** | | |
| STROBE 18 | Summarize key results with reference to study objectives. | p. 10, lines 265–273 |
| STROBE 19 | Discuss limitations, including potential bias or imprecision and their likely direction/magnitude where possible. | p. 12, lines 329–348 |
| RECORD 19.1 | Discuss implications of using routinely collected data, including misclassification, unmeasured confounding, missingness and changing eligibility/data quality. | pp. 11–12, lines 314–348 |
| STROBE 20 | Provide a cautious overall interpretation considering objectives, limitations, multiplicity and other evidence. | pp. 10–13, lines 265–364 |
| STROBE 21 | Discuss generalisability of the study results. | p. 12, lines 329–348 |
| STROBE 22 | State the source of funding and role of funders. | Title page |
| RECORD 22.1 | Describe access to study protocol, raw data and programming code. | p. 8, lines 195–202; Online Resource 3 (supplementary code) |

**B. SAMPL reporting checklist (items applicable to this study)**

SAMPL is a reporting guideline rather than a single numbered checklist. The table maps the SAMPL recommendations relevant to this ecological Bayesian spatiotemporal analysis to the current manuscript.

| **Item** | **Reporting recommendation** | **Where reported in manuscript** |
| --- | --- | --- |
| Analysis purpose | State the purpose of each analysis and distinguish primary from sensitivity analyses. | pp. 3–4, lines 74–76; pp. 6–7, lines 135–194 |
| Variables and transformations | Identify variables, transformations, scaling and derived quantities. | pp. 4–6, lines 99–163 |
| Sample size per analysis | Report the number of observations included in each analysis/model. | pp. 6–8, lines 135–212; Online Resource 2, Tables S1 and S8 |
| Descriptive statistics | Provide descriptive summaries with clear denominators and missing-data handling. | p. 8, lines 206–224; Table 1, p. 16; Online Resource 2, Table S8 |
| Missing data | Describe how missing observations were handled. | p. 8, lines 195–212; p. 12, lines 329–344; Online Resource 2, Table S8, Panels C and E |
| Multiplicity | Describe adjustment or interpretation when multiple statistical tests are performed. | p. 5, lines 124–133; p. 7, lines 189–194 |
| Outliers | Describe identification/exclusion of outliers if performed. | No outlier-based exclusions were performed; prespecified eligibility criteria are reported on p. 6, lines 135–148 |
| Software | Identify statistical software and methods. | p. 8, lines 195–200; Online Resource 3 |
| **REGRESSION / MODEL-BASED REPORTING** | | |
| Model specification | Describe the regression model sufficiently for reproduction, including the equation and likelihood. | p. 6, lines 135–163; Online Resource 2, Table S1 |
| Covariate selection | Describe how predictors were selected. | p. 5, lines 108–123 |
| Interactions | Report interaction analyses or state when none were performed if relevant. | No interaction analysis was prespecified or performed |
| Collinearity | Describe assessment of collinearity when it is relevant to the regression model. | p. 7, lines 182–187; pp. 9–10, lines 257–262; Online Resource 2, Table S6 |
| Effect estimates and uncertainty | Report estimated effects with suitable measures of precision/uncertainty. | p. 9, lines 238–249; Table 2, p. 19, lines 466–478 |
| Model assessment | Describe how model adequacy/predictive performance was assessed. | p. 7, lines 189–194; pp. 9–10, lines 250–262; Online Resource 2, Tables S1, S3, and S7 |
| **BAYESIAN REPORTING** | | |
| Priors specified | Specify prior distributions for model parameters. | p. 6, lines 156–163 |
| Prior rationale | Explain how prior distributions were chosen. | p. 6, lines 156–163; Online Resource 2, Table S4 |
| Posterior summaries | Report posterior central estimates and credible intervals. | p. 9, lines 238–249; Table 2, p. 19, lines 466–478 |
| Prior sensitivity | Assess robustness to plausible alternative priors. | p. 7, lines 179–182; p. 9, lines 250–256; Online Resource 2, Table S4 |

**Guideline references**

Benchimol EI, Smeeth L, Guttmann A, et al.; RECORD Working Committee. The REporting of studies Conducted using Observational Routinely-collected health Data (RECORD) statement. PLoS Medicine. 2015;12(10):e1001885. doi:10.1371/journal.pmed.1001885.

Lang TA, Altman DG. Basic statistical reporting for articles published in biomedical journals: The “Statistical Analyses and Methods in the Published Literature” or the SAMPL Guidelines. International Journal of Nursing Studies. 2015;52(1):5–9. doi:10.1016/j.ijnurstu.2014.09.006.
